# Olfactory Receptor Activation Reduces Platelet Reactivity and Arterial Thrombosis Through Actin Cytoskeleton Remodeling

**DOI:** 10.1161/CIRCULATIONAHA.125.078927

**Published:** 2026-04-07

**Authors:** Anu Aggarwal, Vara Prasad V.N. Josyula, Nancy Wang, Moua Yang, Young Jun Shim, Quinn P. Kennedy, Reina Samuel, Naseer Sangwan, Suman Guntupalli, Matthew Godwin, Huijun Edelyn Park, Mariya Ali, Courtney Jennings, Bhairavi Rajasekar, Alliefair Scalise, Anthony R. Sloan, Justin D. Lathia, Jessica Grondolsky, Sarah M. Schumacher, Shaun Stauffer, Keith R. McCrae, Thomas M. McIntyre, Scott J. Cameron

**Affiliations:** 1 Department of Heart, Blood and Kidney Research, Cleveland Clinic Research, Cleveland Clinic Foundation, OH (A.A., Y.J.S., R.S., N.S., S.G., M.G., H.E.P., M.A., C.J., A.R.S., J.D.L., J.G., S.M.S., K.R.M., T.M.M., S.J.C.).; 2 Center for Therapeutics Discovery, Cleveland Clinic Research , Cleveland Clinic Foundation, OH (V.P.V.N.J., N.W., S.S.).; 3 Section of Vascular Medicine, Department of Cardiovascular Medine, Heart Vascular and Thoracic Institute, Cleveland Clinic Foundation, OH (B.R., A. S, S.J.C.).; 4 Department of Hematology, Taussig Cancer Center, Cleveland Clinic Foundation, OH (S.J.C.).; 5 Division of Hematology and Oncology, Department of Medicine, University of Washington School of Medicine and Bloodworks Northwest Research Institute, Seattle (M.Y., Q.P.K.).; 6 Cleveland Clinic Lerner College of Medicine of Case Western Reserve University, OH (S.J.C.).

**Keywords:** actins, blood platelets, heat-shock proteins, myocardial infarction, receptors, odorant, thrombosis

## Abstract

**BACKGROUND:**

Despite antiplatelet therapy, some patients remain at high ischemic risk because of drug nonresponsiveness or high residual platelet reactivity. We aimed to target an orphan platelet GPCR (G protein–coupled receptor) from the OR (olfactory receptor) family as a novel antithrombotic strategy.

**METHODS:**

Using an engineered reporter cell line expressing human *OR2L13*, an orphan GPCR implicated in limiting platelet reactivity, we conducted a high-throughput screen of 8000 nonodorant bioactive compounds with counterscreen validation. Subsequent studies assessed platelet function in healthy subjects and patients with coronary artery and peripheral artery disease. Phospho-proteomics revealed key signaling pathways, whereas ex vivo and in vivo studies evaluated the impact of a lead compound on platelet signaling, biomechanics, and thrombosis in both arterial and venous vasculature.

**RESULTS:**

We identified 6 OR2L13 (olfactory receptor family 2 subfamily L member 13)-specific agonists that suppressed platelet aggregation and α-granule exocytosis through multiple receptors, suggesting a shared downstream mediator. The lead agonist (CCF0054500) phosphorylated platelet HSP27 (heat shock protein 27), disrupting the actin cytoskeleton and reducing clot retraction (clot area, 70.6 versus 5.2; *P*<0.0001), an effect reversed by HSP27 inhibition. In a murine arterial injury model, CCF0054500 decreased platelet accumulation by 88.9% (*P*<0.0003) without affecting fibrin generation or hemostasis. In a myocardial infarction model with high residual platelet reactivity, CCF0054500 lowered platelet reactivity (*P*<0.0001) and improved left ventricular function (*P*=0.007).

**CONCLUSIONS:**

We describe and characterize the first nonolfactory probe for the purpose of inhibiting platelet activation and thrombosis through downstream HSP27 in a comprehensive investigation using a first-of-its-kind platelet inhibitor targeting an orphan platelet GPCR.

**Clinical Perspective**

**What Is New?**

- A platelet OR (olfactory receptor) was identified as a novel, druggable target for reducing high residual platelet reactivity.
- Platelet OR2L13 activation by a nonodorant chemical probe CCF0054500 broadly inhibits platelet reactivity through PAR1, P2Y_12_, GPVI (glycoprotein VI), and thromboxane receptors, as well as GPIIb/IIIa, uniquely inhibiting both biochemical and biomechanical activation of the platelet.
- CCF0054500 reorganizes the platelet actin cytoskeleton by promoting F-actin depolymerization without diminishing the cytoskeleton globular actin pool, thereby modulating thrombosis-related platelet shape change while preserving physiologic hemostatic function.

**What Are the Clinical Implications?**

- Activating platelet receptor OR2L13 with CCF0054500 lowers high residual platelet reactivity observed in patients with coronary or peripheral artery disease who display significant platelet reactivity despite standard antiplatelet therapy.
- In a murine myocardial infarction model, selective activation of a platelet olfactory receptor by CCF0054500 inhibits high residual platelet reactivity following myocardial infarction and improves cardiac function without impacting the protective mechanism of hemostasis.

Cardiovascular disease remains a leading cause of mortality globally.^1^ Despite Guideline-Directed Medical Therapy, residual cardiovascular risk means that cardiovascular-related deaths continue to rise.^1–4^ Thrombosis is a feared and life-threatening consequence for patients with coronary artery disease (CAD) and peripheral artery disease (PAD). Clinical thrombosis progresses and ends with the activation of platelets by agonists through cell surface receptors (biochemical activation), culminating in thrombus formation. One explanation for residual cardiovascular risk may be the unpredictable behavior of antiplatelet drugs and persistent platelet activation by shear stress (biomechanical activation) that current therapeutics cannot mitigate.^5,6^

Biomechanical platelet activation alters the platelet phenotype and may lead to defects in the protective mechanism of hemostasis while permitting ongoing high residual platelet reactivity (HRPR), which is a risk for thrombosis.^5,7^ In patients with established vascular disease, plaque formation in arteries leads to disturbed blood flow, which causes biomechanical platelet activation. We previously reported that platelet reactivity increases and persists after myocardial infarction (MI) in humans and mice, highlighting an unmet need in the care of patients with thrombotic disease.^8–10^

The OR (olfactory receptor) family of GPCRs (G protein–coupled receptors) in humans represents 18 gene families and 300 subfamilies that encode transmembrane GPCR on the cell surface. Although they are primarily located in sensory neurons of the nasal epithelium, ORs are also known to be expressed ectopically in non-olfactory tissues such as airway smooth muscle, the kidney, colonic and vascular epithelium, and keratinocytes.^11–13^ Ectopic OR expression regulates important physiological and pathological processes, including blood pressure (BP), airway smooth muscle tone, vascular tone and remodeling,^14–18^ which suggests ORs as emerging therapeutic targets for disease. Of the hundreds of ORs identified in humans, we identified only a few in platelet precursor megakaryocytes, and just 3 in healthy adult platelets.^19^ This presents an opportunity for targeted therapy for thrombotic diseases. Among the 3 ORs identified in adult platelets, OR2L13 (olfactory receptor family 2 subfamily L member 13) expression is highest, and it is stored in alpha granules in platelets before membrane surface translocation if platelets are subjected to disturbed blood flow conditions.^19^ A major objective of this study was to evaluate OR2L13 as a druggable target using a high-throughput screen and investigate the hitherto undiscovered olfactory signal transduction pathways in platelets to limit thrombosis through mechanisms unreachable by current antiplatelet drugs.

## METHODS

For detailed information on mouse experimental procedures, refer to the Supplemental Material. Primary data, where useful to the general scientific community, are uploaded as supplemental files. The corresponding author can be contacted for any data or reagents used with the appropriate regulatory request documents.

### Human Subjects

All healthy subjects were recruited only after informed consent by a coordinator not involved in clinical care using the following protocols: 19-1451 for patients and 20-413 for healthy volunteers. Patients with CAD and PAD were recruited from the clinic for the validation of the results. Biobanks for isolation of blood and studying platelets from healthy individuals and patients with vascular disease are registered on ClinicalTrials.gov as NCT05628948 and NCT05628974, respectively. All study procedures were conducted in accordance with the Declaration of Helsinki and our approved local institutional review board protocol at Cleveland Clinic.

### Experimental Animals

Murine protocols were approved by the Institutional Animal Care and Use Committee at Cleveland Clinic. Male and female wild-type FVB/NJ (Friend Virus B NIH Jackson) mice 6 to 8 weeks old were used in this study (The Jackson Laboratory, Bar Harbor, ME). Retro-orbital blood collected into heparinized Tyrode’s solution, as described by us previously, was used for platelet isolation and to keep platelets in a silent state before agonist stimulation.^10^ Complete blood counts were determined from retro-orbital blood collected into EDTA tubes and analyzed using an Abaxis HM5C automated complete blood analyzer (Allied Analytic, LLC, Tampa, FL). The sample size for all mouse experiments was determined by power calculation. All animal experiments in which a drug was compared with placebo were conducted in a manner blinded to the experimentalist. Animals were monitored twice daily by investigators, trained veterinary technicians and, when needed, by licensed veterinarians to assess distress, illness, and pain.

### Statistics

Data are presented as the mean ± SEM or mean ± SD depending on distribution and population size unless stated. Normality was evaluated by the Shapiro-Wilk test. For normally distributed data between 2 comparative groups, a 2-tailed Student *t* test was used. For nonnormally distributed data, the Mann-Whitney U test was used. For Gaussian-distributed data in 3 or more groups, 1-way ANOVA followed by Bonferroni multiple comparison test was used. Otherwise, the Kruskal-Wallis test followed by Dunn post-hoc test correction was used. A mixed-effects model (Restricted Maximum Likelihood) with Geisser-Greenhouse correction followed by Tukey multiple comparison test was used for analysing the effect of two categorial independent factors on a single continuous dependent factor when data is colleced from same animal over time. Two-way ANOVA with Šidák multiple comparisons test was also used. Significance was accepted as a *P* value of <0.05. Analyses were conducted using GraphPad Prism 10 (GraphPad Software).

## RESULTS

### Nonodorant Compounds Activate OR2L13

**Figure 1.**
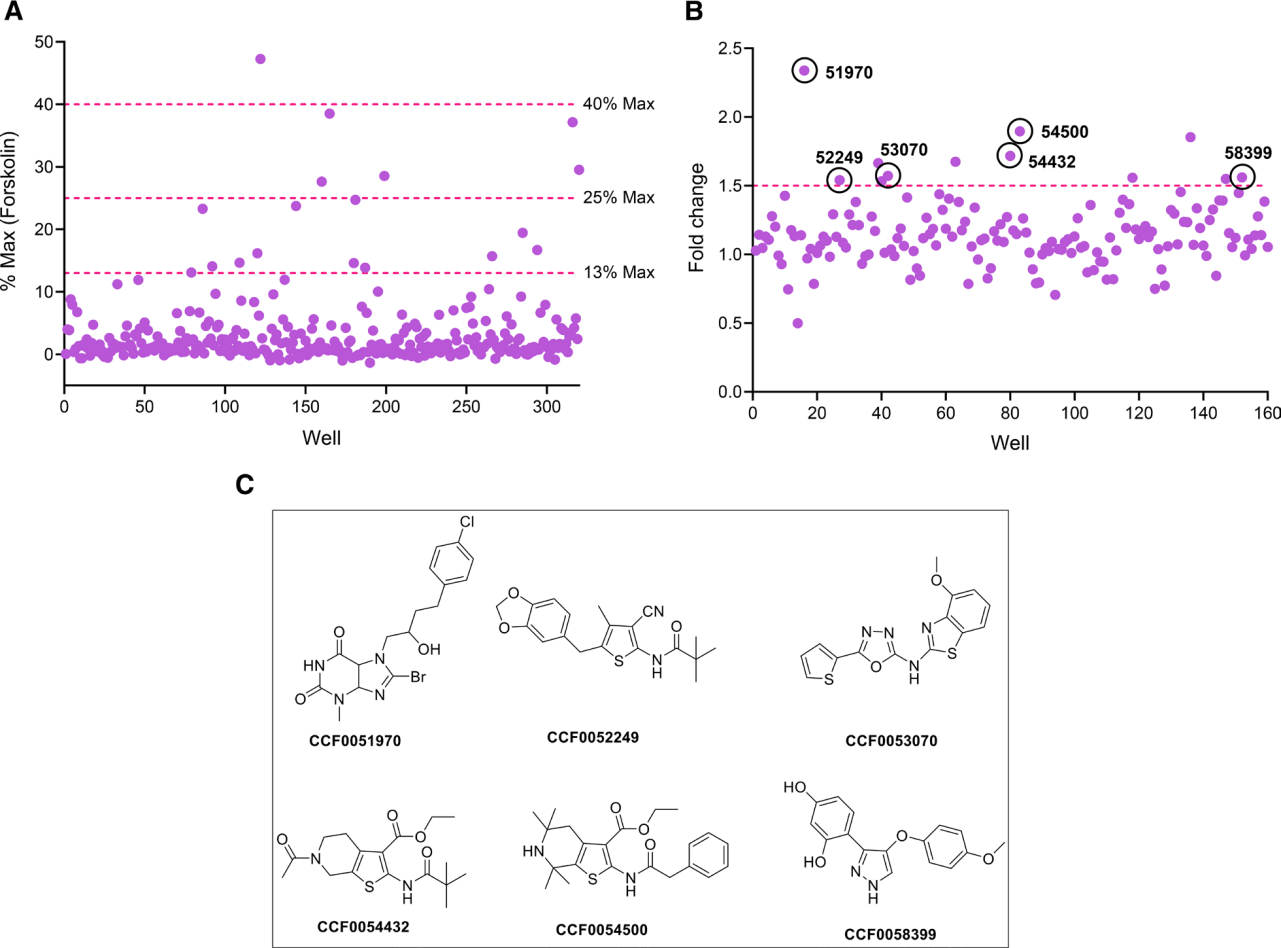
**Screening of an 8000 bioactive small molecule library for nonodorant OR2L13 agonists. A**, Primary screen. A total of 8,000 nonolfactory compounds were screened at 50 μM concentration using a OR2L13 HEK293 cAMP-producing reporter cell line. A total of 169 hits (2.1% hit rate) >25% threshold of % maximum forskolin were determined. All plates passed a Z-prime >0.5. Each plate had 500 μM L-carvone as a positive control for OR2L13 receptor and 3 μM forskolin as a positive control for adenylyl cyclase. **B**, Counterscreen using a HEK293 cAMP-producing reporter cell line was set up. Fold change of 169 hits was determined as the ratio of luminescence of OR2L13 HEK293 cAMP/HEK293 cAMP reporter cell lines. Hits >1.5-fold change above baseline (CCF0054500, CCF0054432, CCF0053070, CCF0052249, CCF0051970, and CCF0058399, each 50 μM final concentration) encircled in black were selected for further analysis. **C**, Molecular structure of lead OR2L13 agonists. The chemical structure of each lead compound is indicated with compound identifier below. Max indicates maximum; OR, olfactory receptor; and OR2L13, olfactory receptor 2L13.

We previously reported that OR2L13 activation in platelets increases cAMP (cyclic adenosine monophosphate) through odorant stimulation by a molecule that is the active ingredient in spearmint.^19^ Using an engineered reporter cell line activated by OR agonists, cAMP production is readily detectable upon OR2L13 stimulation. We screened a library of nonodorant bioactive compounds known to activate GPCRs to identify and test a new approach for antiplatelet therapy. The 8000 nonolfactory compounds bioactive library screen identified 169 preliminary hits (2.1% hit rate, 50 μM concentration), exceeding the 25% threshold Z-prime (the statistical separation between positive and negative controls in an assay) that was >0.5 for each plate in the high-throughput screen. To limit variability due to cell passaging, data were normalized to forskolin in each plate (positive control; directly activates adenylyl cyclase to synthesize cAMP). In a counterscreen at 50 μM concentration with a vector HEK293-CRE (human embryonic kidney 293-cAMP response element) cell line, we validated the specificity of initial hits for OR2L13. Twelve compounds emerged as agonists for OR2L13 (CCF0051970, CCF0054500, CCF0057537, CCF0054432, CCF0053625, CCF0052884, CCF0053070, CCF0058399, CCF0056873, CCF0052249, CCF0058334, and CCF0053066). Each of the 12 compounds demonstrated reporter luminescence >1.5-fold above baseline (Figure 1A and 1B). Of those 12 compounds, only 6 were available for commercial synthesis and use. The EC_50_ for each of the 6 OR2L13 specific agonists was determined (CCF0054500, CCF0054432, CCF0053070, CCF0052249, CCF0051970 and CCF0058399) (Figure S1). Chemical structures for the 6 lead compounds are shared in Figure 1C. Given that OR2L13 is a GPCR, 6 ligands were further evaluated using dynamic mass redistribution assay in a dose-response manner (6.25 μM–50 μM). We found significantly higher GPCR responses at all compound doses compared with vehicle (Figure S2). Collectively, these data validated 6 leading nonodorant ligands as agonists for human OR2L13 expressed on platelets.

### Effect of Nonodorant Ligands of OR2L13 on Platelet Function

**Figure 2.**
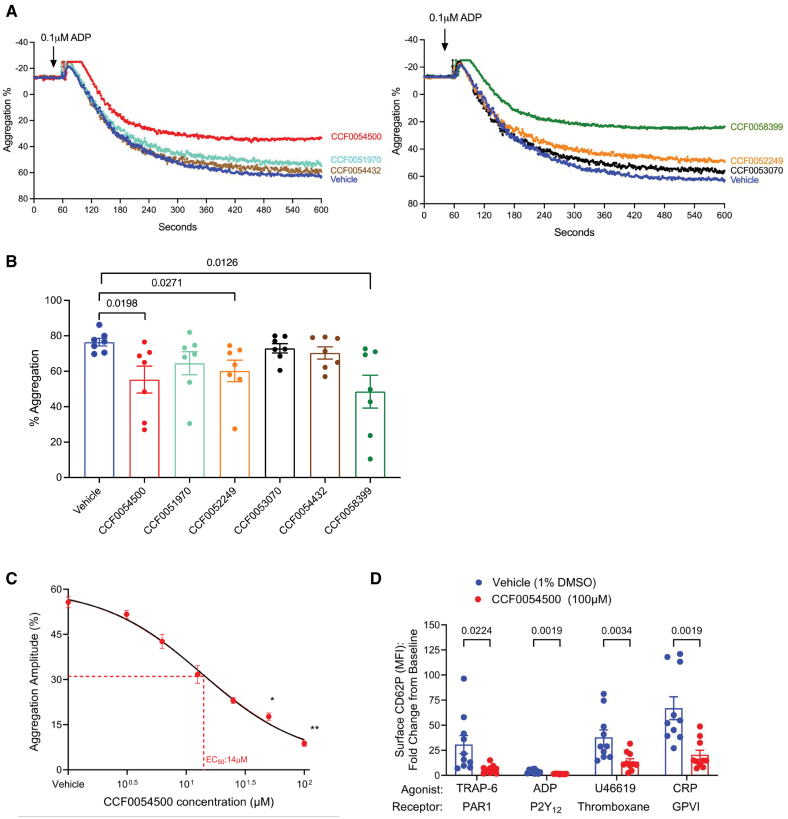
**Impact of nonodorant OR2L13 agonists on platelet activation. A**, Light transmission aggregometry for the top OR2L13 agonists (CCF0054500, CCF0051970, CCF0052249, CCF0053070, CCF0054432, and CCF0058399) using platelet-rich plasma (PRP) from healthy subjects (n=7). PRP was preincubated with 100 μM ligand or vehicle (1% DMSO) for 30 minutes at 37 °C and then was run on an aggregometer. Baseline was determined for 30 s, and then 0.1 μM ADP was added to determine the platelet aggregation. Representative plot of light transmission aggregation with top 6 hits in comparison to vehicle are shown. **B**, Summarized aggregometry data is shown as mean percent maximum aggregation ± SEM, n=7 independent healthy volunteers in each group. *P* value was determined using 1-way ANOVA. **C**, Light transmission aggregometry dose response using the OR2L13 agonist CCF0054500 (1–100 μM) added to PRP from healthy controls. PRP was preincubated with increasing concentrations of CCF0054500 (1–100 μM) or vehicle (1% DMSO) for 30 minutes at 37 °C, and platelet aggregation was noted after activation by 0.1 μM ADP. Summarized aggregometry data are shown as mean percent aggregation ± SEM (n=3 independent healthy volunteers in each group; each performed in duplicate). The EC_50_ of CCF0054500 for platelet inhibition is indicated by the red line and calculated using nonlinear fit. Statistical analysis was performed using the Kruskal-Wallis test with Dunn multiple comparisons test when calculating change from vehicle. **D**, Effect of CCF0054500 on platelet alpha granule exocytosis. Changes in surface P-selectin expression of washed platelets treated with CCF0054500 (100 μM) and vehicle (1% DMSO) for 30 minutes at 37 °C, followed by activation with TRAP-6 (10 μM), ADP (10 μM), U46619 (5 μM), and CRP (0.5 μg/mL). The results are expressed as mean fluorescence intensity of P-selectin from baseline ± SEM (n=10; each performed in quadruplicate; 2-way ANOVA). Data from platelet reactivity of CCF0058399 and CCF0052249 in (Figure S3). CRP indicates collagen-related peptide; DMSO, dimethyl sulfoxide; GPVI, glycoprotein VI; MFI, mean fluorescence intensity; OR, olfactory receptor; and TRAP-6, thrombin receptor activator peptide 6.

The efficacy of the 6 leading nonolfactory compounds to inhibit platelet function was determined in platelet-rich plasma (PRP) and in washed platelets. Initially, the platelet inhibitory effect of the 6 lead compounds was assessed in PRP using light transmission aggregometry. Plasma albumin can bind to compounds and reduce their effective concentration, so PRP was first used to evaluate the efficacy of each compound. PRP was preincubated with the 6 lead compounds (CCF0054500, CCF0054432, CCF0053070, CCF0052249, CCF0051970, and CCF0058399) for 30 minutes at room temperature followed by stimulation with ADP (adenosine diphosphate; 0.1 μM). Only 3 compounds (CCF0054500, CCF0052249, and CCF0058399) inhibited ADP-mediated platelet aggregation in healthy subjects beyond 30% (Figure 2A and 2B). The EC_50_ for CCF0054500 to inhibit platelet aggregation in PRP from healthy subjects after ADP-mediated activation was found to be in the low micromolar range (14 μM) (Figure 2C). Next, antiplatelet properties of the 3 leading OR2L13 compounds were further evaluated by measuring surface CD62P (P-selectin) expression using flow cytometry. Evaluating platelet activation by α-granule exocytosis interrogates multiple platelet receptor–mediated activation pathways simultaneously (PAR1 [ Proteinase-activated receptor 1], P2Y_12_, thromboxane receptor, and GPVI [glycoprotein VI]). CCF0052249 and CCF0058399 did not suppress platelet activation through any of the common pathways (Figure S3). Surprisingly, CCF0054500 inhibited agonist-mediated platelet activation (TRAP-6 [thrombin receptor activator peptide 6] 10 μM, ADP 10 μM, U46619 5 μM, and CRP [collagen-related peptide] 0.5 μg/mL) through all receptors assessed, including Gαq, Gαi, as well as GPVI, suggesting a noncanonical downstream signaling mediator previously undiscovered in platelet ORs (Figure 2D). CCF0054500 was therefore progressed as a promising nonodorant chemical probe for OR2L13 because of its inhibitory effect on platelets in both PRP and washed platelets.

### OR2L13 Agonist Inhibits In Vivo Thrombus Formation Under Shear Stress Without Affecting Coagulation

**Figure 3.**
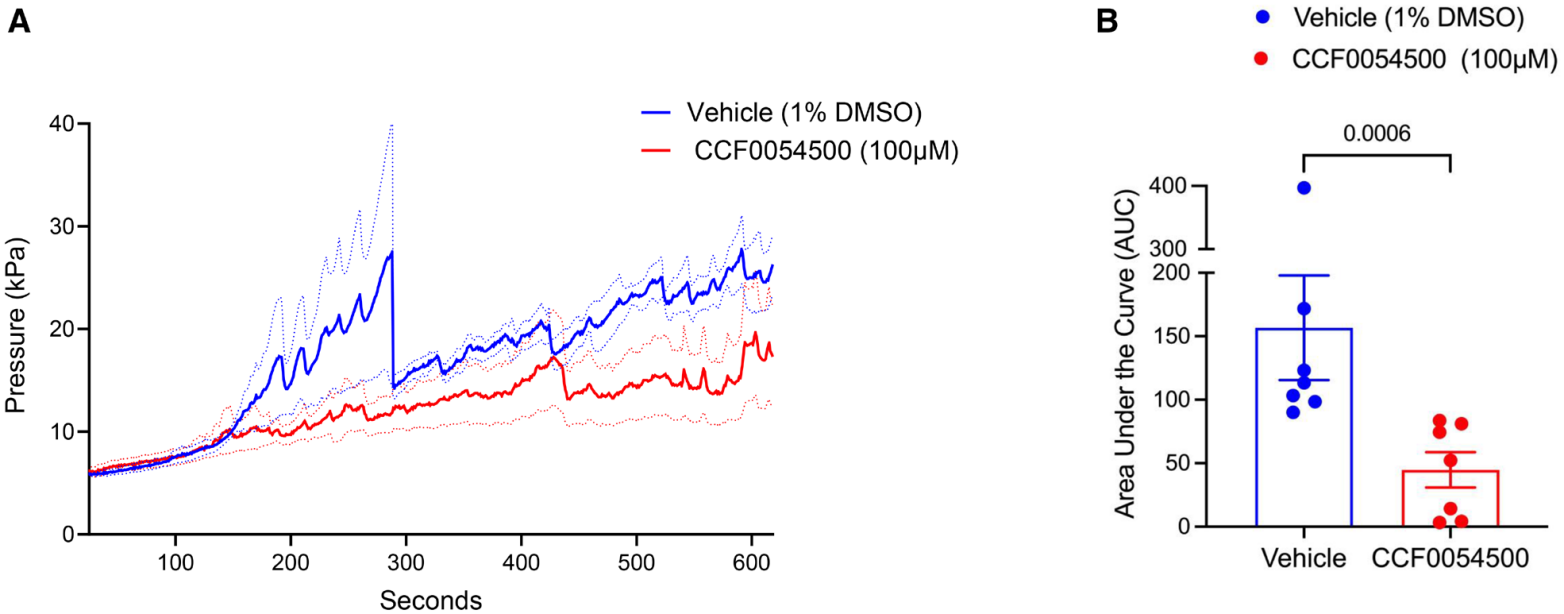
**CCF0054500 through OR2L13 inhibits biomechanical platelet activation. A**, Total Thrombus-formation Analysis System (T-TAS01) evaluates thrombosis of whole blood under high arterial shear stress in a microfluidics system (1500 s^-1^) as a function of time and peak pressure. Data are represented as mean ± SEM (broken lines). CCF0054500 (100 μM) decreases occlusion under shear stress of 1500 s^-1^ determined in healthy subjects (n=7). Data summarized as (**B**) area under the curve as mean ± SEM. *P* value was determined by nonparametric Mann-Whitney test. DMSO indicates dimethyl sulfoxide; and OR, olfactory receptor.

To assess the effect of CCF0054500 on thrombosis under more pathological conditions, CCF0054500 was introduced into whole blood from healthy subjects using the Total Thrombus-Formation Analysis System (T-TAS01), in which blood is passed through collagen-coated microfluidics channels under varying magnitudes of shear stress. CCF0054500 (100 μM) decreased thrombosis in whole blood by >50% compared with vehicle, uniquely under conditions of high arterial shear rate (1500 s^-1^; Figure 3), but not lower arterial shear rate (600 s^-1^; Figure S4). Because CCF0054500 inhibited thrombosis in whole blood, isolated washed platelets, and PRP from humans, we next prioritized assessing the impact of CCF0054500 on the coagulation cascade, anticipating potential bleeding as a side effect. Platelet-poor plasma was used to assess real-time fibrin (factor Ia) formation, which is the most downstream component of the coagulation cascade. We did not observe any impact of CCF0054500 on fibrin formation in platelet-poor plasma isolated from healthy humans when assessed by the rate of clot growth ex vivo or absolute clot size (Figure S5).

### OR2L13 Agonist Inhibits Arterial Thrombosis In Vivo

**Figure 4.**
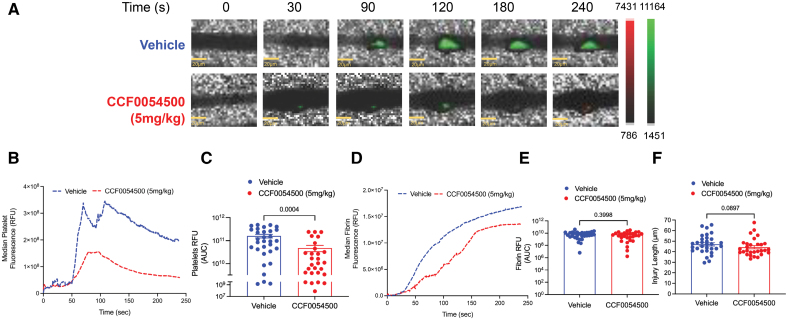
**CCF0054500 decreases platelet accumulation in vivo in an arterial thrombosis model. A**, Intravital microscopy images of the mouse cremaster arterioles showing platelet (green) and fibrin (red) accumulation after laser injury in vehicle and CCF0054500 (5 mg/kg for 3 consecutive days intraperitoneally) treated groups. Yellow scale bar, 20 μm. **B**, Median integrated fluorescence intensity of platelets over time. **C**, Quantification of the normalized platelet accumulation shown as mean ± SEM. **D**, Median integrated fluorescence intensity of fibrin accumulation over time. **E**, Quantification of normalized fibrin accumulation shown as mean ± SEM. **F**, Injury size differences between vehicle and CCF0054500. Vehicle, n=31 injuries between 4 mice; CCF0054500, n=29 injuries between 3 mice. Data are represented as mean ± SEM. *P* values were determined by nonparametric Mann-Whitney test. AUC indicates area under the curve; and RFU, relative fluorescence unit.

To ascertain the bioavailability of CCF0054500 and to assess cross-species efficacy, the compound was introduced into FVB/NJ (Friend Virus B NIH Jackson) mice, known to express high quantities of the human OR2L13 ortholog Olfr168 in platelets,^19^ by daily intraperitoneal injections. This permits the assessment of CCF0054500 on hemostasis and occlusive thrombosis in vivo. Laser injury–initiated thrombus formation in the mouse cremaster vasculature was measured by platelet and fibrin accumulation at the blood vessel intima over time after consecutive daily doses of CCF0054500 (5 mg/kg daily intraperitoneally). CCF0054500 decreased platelet accumulation at the vessel intima by a log-fold magnitude (88.9%) compared with the vehicle control treatment group (Figure 4A through 4C). Consistent with findings in human plasma ex vivo (Figure S5), fibrin formation in mice was unaffected by CCF0054500 (*P*=0.39; Figure 4D and 4E), suggesting no impact of CCF0054500 on the coagulation cascade in vivo. The median arterial injury sizes were similar between the 2 treatments (Figure 4F; Videos S1 and S2), indicating that the differences observed were a result of the OR2L13/Olfr168 chemical probe. These data suggest that CCF0054500 limits thrombosis by inhibiting platelet accumulation during thrombus formation without an impact on fibrin formation.

### OR2L13 Agonist Inhibits Thrombosis In Vivo Under Conditions of Vascular Obstruction

**Figure 5.**
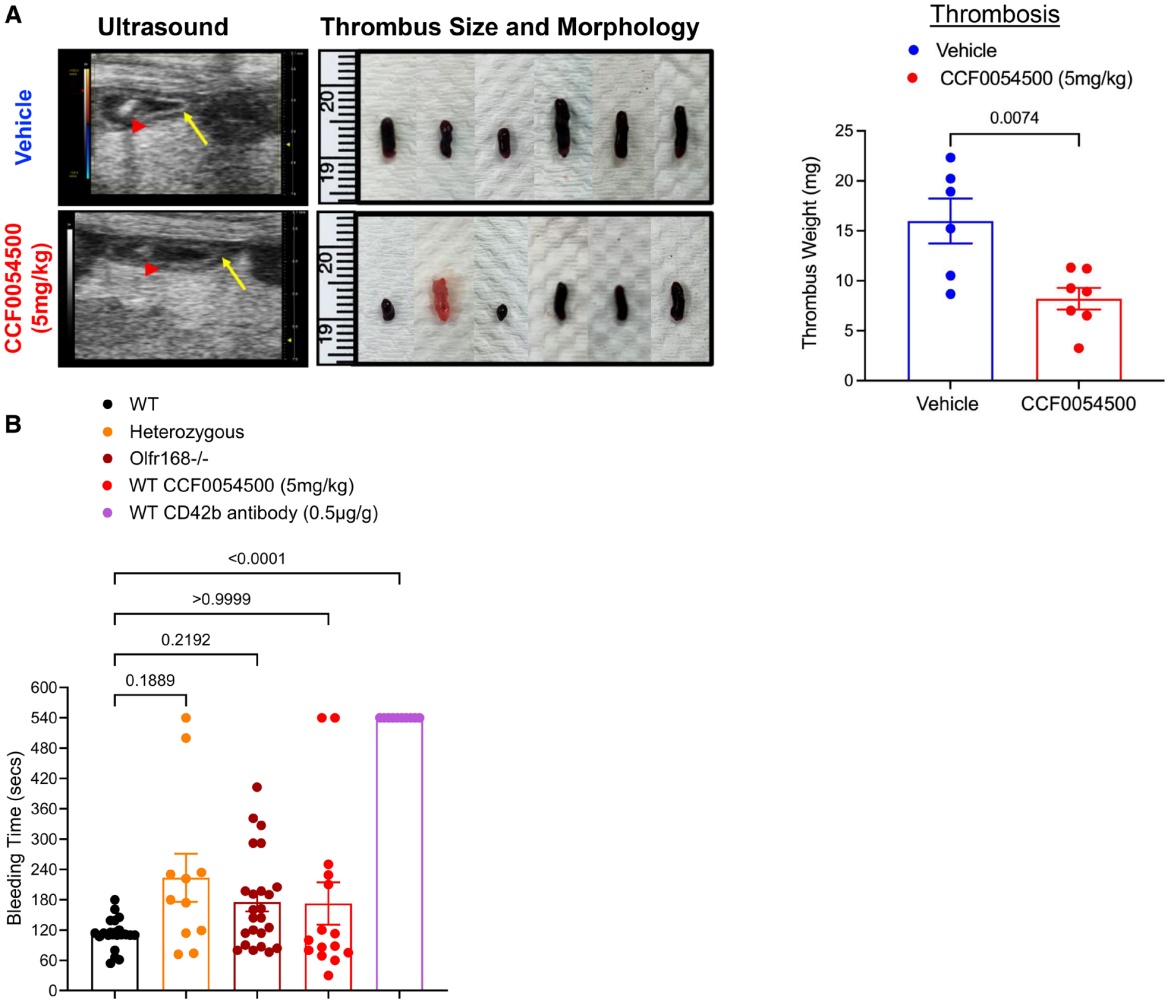
**CCF0054500 decreases thrombus size in vivo in an inferior vena cava stasis model. A**, Smaller thrombus was detected by B-mode ultrasound (red arrowhead) in the group treated with CCF0054500 (5 mg/kg per day for 3 days intraperitoneally) than in the vehicle-treated group following inferior vena cava (IVC) constriction (yellow arrow, the region of ligature). Thrombus weight is represented as mean ± SEM (n=6 or 7, *t* test). **B**, CCF0054500 does not alter hemostasis. Wild-type (WT) mice were treated with CCF0054500 (5 mg/kg per day for 3 days intraperitoneally) and bleeding time assessed after tip amputation (n=16). Bleeding times were compared within different groups; WT, n=22; heterozygous, n=11; *Olfr168*^-/-^, n=24; mice treated with anti-CD42b (glycoprotein Ibα) antibody, 0.5 μg/g daily for 3 days intraperitoneally, n=10. Data are represented as mean ± SEM (n=10–24). *P* value was determined using 1-way ANOVA.

Mechanical stress on blood leads to thrombosis in vivo and is readily assessed by inferior vena cava constriction, which creates disturbed blood flow proximal to the suture line and exposes blood to external mechanical forces as well as stasis.^20^ FVB/NJ mice were treated with CCF0054500 (5 mg/kg daily intraperitoneally) followed by inferior vena cava constriction. CCF0054500-treated mice exhibited a 49% reduction in average thrombus size compared with vehicle-treated mice (thrombus weight, 8.20±2.6 mg versus 15.98±5.0 mg; *P*=0.0074) (Figure 5A). Thrombus composition of cells, including red blood cells (CD235a), platelets (CD41), white blood cells (CD45), and fibrin, was comparable between CCF0054500-treated and vehicle-treated mice (Figure S6). Baseline blood counts in mice were unchanged after CCF0054500 treatment, illustrating the absence of toxic effects on hematopoiesis (Figure S7). Last, CCF0054500 did not affect hemostasis given the duration of bleeding in CCF0054500-treated mice was markedly shorter than in platelet-depleted mice (anti-CD42b treatment, >9 minutes). Platelet count decreased in anti-CD42b treated mice from 555×10^9^/L pretreatment to 8.1×10^9^/L post-treatment (*P*<0.0001). Moreover, bleeding times in CCF0054500-treated mice were comparable to those in FVB/NJ wild-type, heterozygous (*Olfr168*^-/+^), or knockout (*Olfr168*^-/-^) mice, further suggesting no impact of CCF0054500 on hemostasis (Figure 5B).

### CCF0054500 Decreased Platelet Reactivity and Improved Heart Function After MI

**Figure 6.**
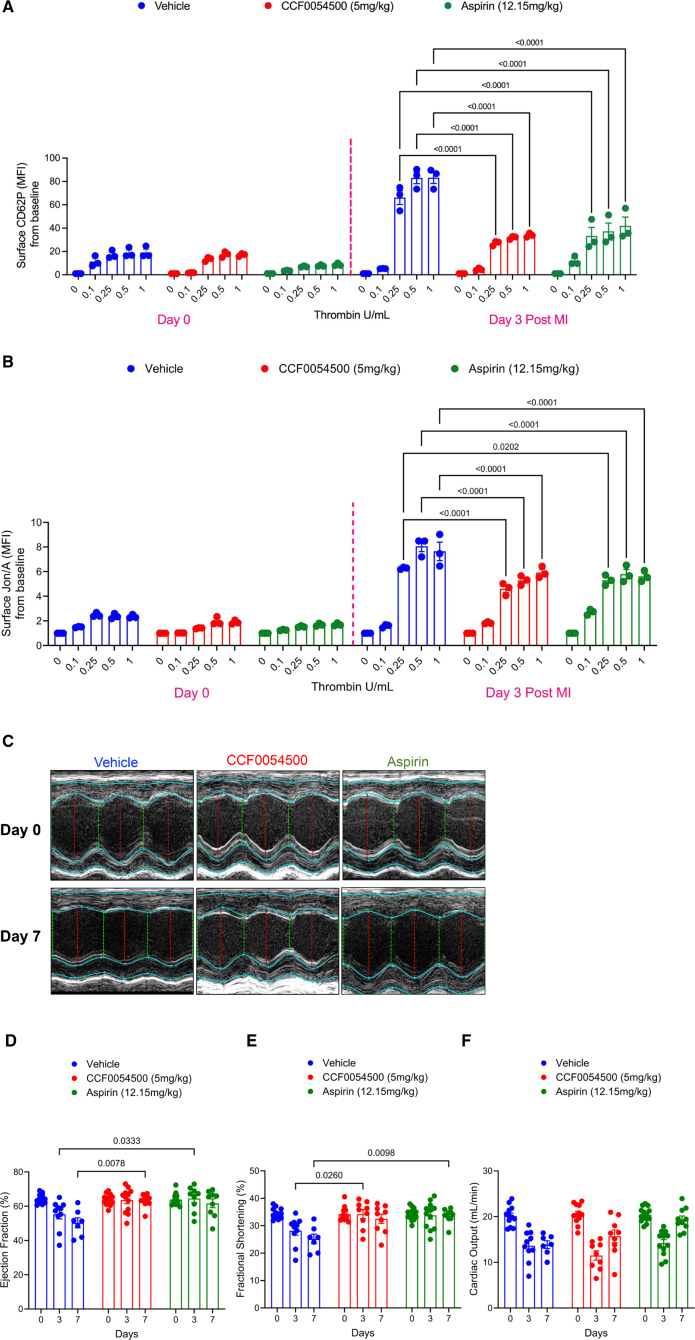
**Effect of CCF0054500 on platelet reactivity and cardiac function after myocardial infarction in mice.** Left anterior descending (LAD) coronary artery of FVB/NJ mice was ligated to create a transmural myocardial infarction. Then these mice were treated with vehicle (75% DMSO), CCF0054500 (5 mg/kg per day), or aspirin (12.15 mg/kg per day) intraperitoneally daily for 7 days. Blood was collected at day 0 (before the LAD ligation) and day 3 after MI from the retro-orbital sinus. **A**, Platelets were isolated and activated with different doses of thrombin (0.1, 0.25, 0.5, and 1 U/mL) for 15 minutes and then incubated with CD62P antibody for 30 minutes at room temperature. Results are expressed as Mean Fluorescence Intensity of CD62P from the baseline (n=4, Two-way ANOVA). Changes in surface CD62P expression provides information about alpha granule exocytosis. **B**, Platelets were isolated and activated with different doses of Thrombin (0.1, 0.25, 0.5 and 1U/mL) for 15 minutes and then incubated with Jon/A antibody for 30 minutes at room temperature. Results are expressed as Mean Fluorescence of Jon/A from the baseline (n=4, Two-way ANOVA). Jon/A binds to active form of GPIIb/IIIa and surface Jon/A expression provides information about platelet aggregation. **C**, Representative echocardiography M-mode images for each group are shown. Quantitative assessment of myocardial function after MI and the impact of CCF0054500 was determined by measuring: **D**, Ejection fraction (EF), **E**, Fractional shortening (FS) and **F**, Cardiac output (CO) at day 0, day 3 and day 7 after MI. Data are displayed as mean SEM, n=9-12 in each group. Statistical significance was determined using a two-way ANOVA with a mixed-effects model (REML) and the Geisser-Greenhouse correction followed by Tukey’s multiple comparisons test with individual variance computed for each comparison. DMSO, indicates Dimethyl sulfoxide; FVB, (Friend Virus B NIH Jackson); GPIIb/IIIa, Glycoprotein IIb/IIIa; MFI, Mean Fluorescence Intensity; MI, myocardial infarction; and REML, Restricted Maximum Likelihood.

Antiplatelet therapy remains a cornerstone in the management of acute coronary syndrome, significantly reducing thrombotic risk and lowering the incidence of major clinical events such as MI and death.^21,22^ Platelet activation and aggregation in response to endothelial injury are central to the pathophysiology of acute coronary syndrome, and current antiplatelet agents target distinct platelet receptors to mitigate these events. Antiplatelet therapy is a key component of secondary prevention after ST‑segment–elevation MI, but it has been shown to provide only partial protection in patients with chronic CAD. To determine whether CCF0054500 exerts antiplatelet effects comparable to established therapies, we assessed platelet activation before and after acute transmural MI of the left ventricle in a murine model, in which MI was not rescued. Properties of platelets were studied in the post-MI environment in which CCF0054500 was given in vivo. In vehicle-treated mice, platelet reactivity—measured by surface CD62P expression (platelet activation by α granule exocytosis)—remained significantly elevated on day 3 after MI. In contrast, CCF0054500 treatment markedly attenuated thrombin-induced CD62P expression (T0.25U/mL=27.10±2.0; T0.5U/mL=31.82±1.37; T1U/mL=33.91±1.69; *P*<0.0001), comparable to (and slightly better than) platelet inhibition observed with aspirin (T0.25U/mL=33.26±12.5; T0.5U/mL=37.23±12.28; T1U/mL=41.92±13.11; *P*<0.0001), an established antiplatelet agent in MI (Figure 6A). Similarly, surface Jon/A antibody binding (platelet activation by glycoprotein IIb/IIIa) was significantly increased in vehicle-treated mice after MI in response to increasing concentrations of thrombin. In contrast, CCF0054500 treatment markedly attenuated thrombin-induced Jon/A binding (T0.25U/mL=4.60±0.49; T0.5U/mL=5.29±0.38; T1U/mL=5.92±0.46; *P*<0.0001), comparable to (and slightly better than) the reduction observed with aspirin (T0.25U/mL=5.30±0.41; T0.5U/mL=5.79±0.67; T1U/mL=5.61±0.44; *P*<0.0001) (Figure 6B). Cardiac function after MI was assessed by echocardiography in vehicle-, CCF0054500-, and aspirin-treated mice. Representative M-mode images for each group are shown, with progressive left ventricle dilation after MI that was prevented by CCF0054500 in a similar manner to aspirin (Figure 6C). Myocardial systolic performance assessed by ejection fraction declined progressively in the vehicle-treated group from baseline (day 0, 64.32%±0.79%) to day 3 (55.25%±2.68%) and day 7 after MI (50.30%±2.92%). In contrast, ejection fraction after MI was preserved in mice treated with CCF0054500 (day 0, 63.77%±0.83%; day 3, 63.51%±2.03%; day 7, 63.17%±1.17%; *P*=0.0078) and aspirin (day 0, 63.80%±1.04%; day 3, 64.47%±2.40%; *P*=0.033; day 7, 61.69%±2.55%) (Figure 6D).

Similarly, myocardial systolic performance assessed by fractional shortening declined in vehicle-treated mice after MI. In contrast, CCF0054500-treated mice exhibited significantly preserved fractional shortening after MI, reaching 121% of vehicle-treated values at day 3 after MI (34.20%±1.64% versus 28.18%±1.62%; *P*= 0.0260) and 130% at day 7 after MI (32.52%±1.67% versus 25.16%±1.75%; Figure 6E), which was comparable to the aspirin-treated groups (120% at day 3 and 133% at day 7). In additional supportive data, cardiac output on day 7 after MI in the CCF0054500-treated group showed a 140% increase compared with vehicle-treated mice (19.36 mL/min±0.78 versus 13.87 mL/min±1.06; Figure 6F). Additional measures of left ventricle dimensions and volumes are provided (Figure S8A). Consistent with the improvement in cardiac function, treatment with CCF0054500 resulted in 100% post‑MI survival compared with 75% survival in aspirin‑treated mice and 37.5% in vehicle‑treated controls in a limited observational cohort (Figure S8B). Intriguingly, platelet surface expression of Olfr168 (murine ortholog of OR2L13) increased after MI (Figure S8C), potentially enhancing the binding of CCF0054500. Treatment with CCF0054500 did not affect circulating blood cell populations after MI (Figure S8D), suggesting the chemical probe is not toxic to bone marrow cellular development. Mild blood loss anemia was noted in aspirin-treated mice compared with CCF0054500-treated mice after MI, with a decrease in blood hemoglobin with aspirin only, consistent with our previous studies showing platelet OR2L13-modulating compounds do not adversely affect hemostasis.

### CCF0054500 Activates Platelet HSP27 and Depolymerizes the Filamentous Actin Cytoskeleton

**Figure 7.**
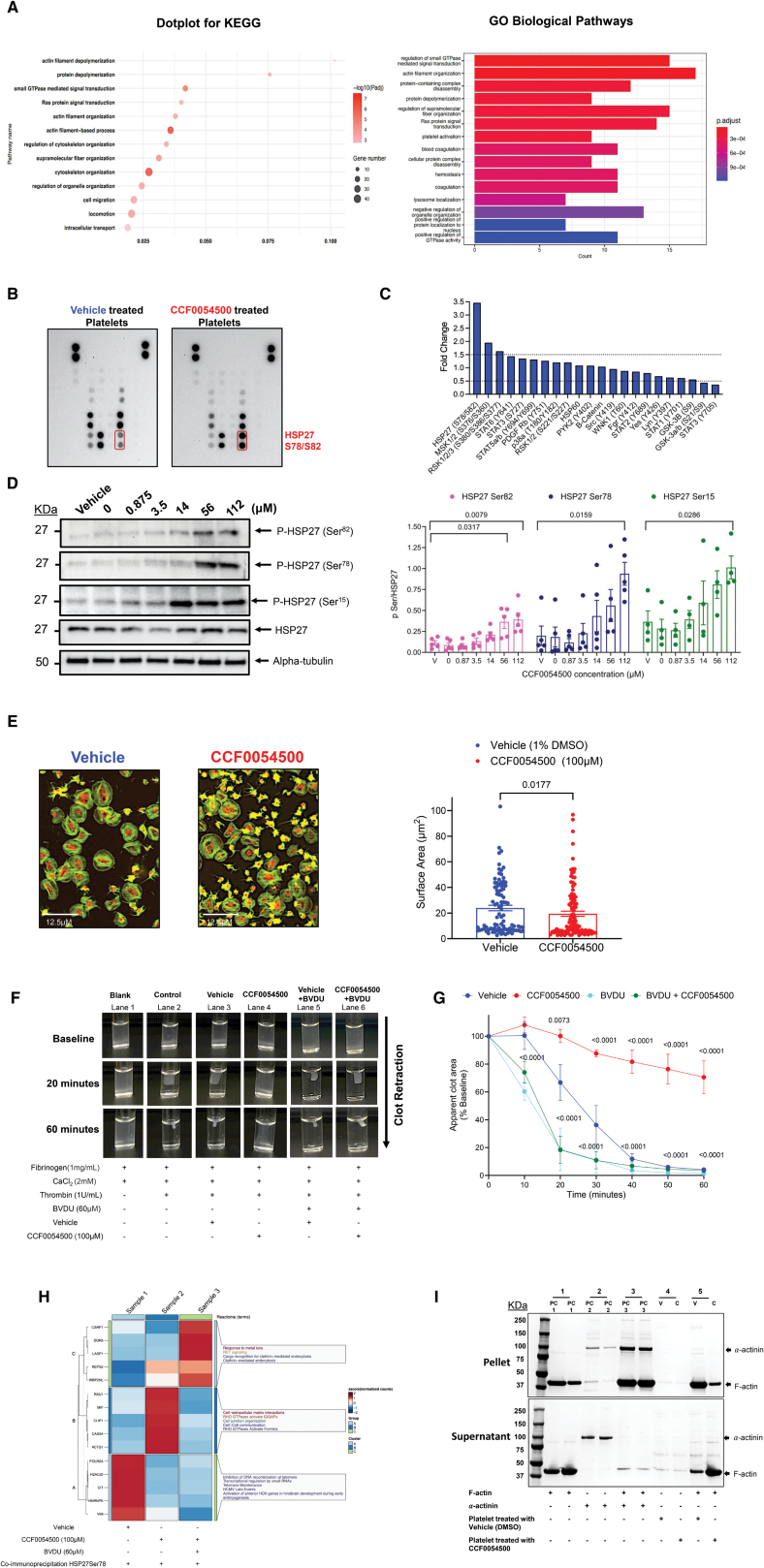
**CCF0054500 rearranges the platelet actin cytoskeleton through HSP27. A**, Treatment of healthy human platelets with 100 μM CCF0054500 versus vehicle and unbiased phosphoproteomics and protein identification by mass spectrometry revealed drug-induced changes in the platelet actin cytoskeleton and rearrangement of intracellular transport by Gene Ontology (GO) analysis with enrichment shown by Kyoto Encyclopedia of Genes and Genomes (KEGG) display. Washed platelets isolated from n=3 healthy subjects. **B**, Treatment of human platelets with 100 μM CCF0054500 and vehicle (DMSO) for targeted phosphokinase array revealed marked phosphorylation of heat shock protein 27 (HSP27). **C**, Densitometry of CCF0054500/vehicle-treated platelets for each phosphoprotein (n=3). **D**, Platelet lysates were prepared from platelets treated with different doses of CCF0054500 (0, 0.875, 3.5, 14, 56 and 112μM) or vehicle for 30 minutes, then separated by SDS-PAGE. HSP27 phosphorylation at serine 15, 78, and 82 was validated by immunoblotting. To ensure equal loading, the membranes were re-probed with anti-alpha tubulin antibody. The intensity of the signals was determined by densitometry, and values shown as the ratio of pHSP27/HSP27 for n=4-5 healthy subjects. *P* value was determined using Two-way ANOVA **E**, Spreading of platelets on a fibrinogen matrix to show actin cytoskeleton dynamics (green=filamentous actin, red=P-selectin). Surface area quantified using Image J from 42 random fields per subject (n=3). Data is represented as mean ± SEM, *P* value was determined by t-test. White scale bar=12.5µm. **F**, Morphological change of clot over time with washed platelets from healthy subjects treated with vehicle or CCF0054500 (100μM) *ex vivo*: Different tubes were set up including blank (without thrombin, Lane 1), control (Lane 2), washed platelets treated with Vehicle (Lane 3) and 100μM CCF0054500 (Lane 4) for 30 minutes at 37ºC. In lane 5 and 6, washed platelets were pre-treated with 60μM BVDU (HSP27 inhibitor) for 45 minutes at 37ºC and then were treated with vehicle or 100μM CCF0054500 for 30 minutes at 37ºC. Then CaCl_2_ (2mM), Thrombin (1U/mL) and Fibrinogen (1mg/mL) were added, and images were taken every 10 minutes until 60 minutes. **G**, Graphical representation of clot retraction at different time points. Statistical significance was evaluated with Two-way ANOVA with *P* value as noted. Data are expressed as mean ± SEM between vehicle (DMSO) vs CCF0054500 and CCF0054500 vs BVDU+CCF0054500 (n=5). **H**, Co-immunoprecipitation of platelets treated with vehicle, CCF0054500 or CCF0054500+BVDU (HSP27 inhibitor) was performed using a HSP27-Ser78 antibody. Reactome pathway analysis of cellular processes of proteomic LC-MS/MS data revealed that CCF0054500 activates Rho GTPases-mediated cytoskeletal rearrangement, an effect that was suppressed by BVDU. **I**, Filamentous-actin (F-actin) binding assay performed on washed platelets treated with vehicle or CCF0054500 (100µM). Pellet and supernatant fractions were then collected for each reaction and separated by 4-20% SDS-PAGE and stained with 0.1% Coomassie blue. Lane 1, F-actin (positive control 1). Lane 2, α-actinin (positive control 2). Lane 3, α-actinin and F-actin (positive control 3). Lane 4, Test sample alone (negative control). Lane 5, Test sample and F-actin. In Lane 5, in the presence of F-actin, there is less test protein in the pellet for CCF0054500-treated, washed platelets than vehicle but more in supernatant, suggesting depolymerization of F-actin with CCF0054500. BVDU indicates brivudine; C, CCF0054500; DMSO, Dimethyl sulfoxide; GTPase, Guanosine Triphosphatases; LC-MS/MS, Liquid chromatography- mass spectrometry; Padj, adjusted *P* value; PC1-3, Positive control 1-3; pHSP, phosphorylated heat shock protein; and V, vehicle.

Platelet activation almost always activates a cascade of protein kinases and causes protein phosphorylation events that alter platelet reactivity.^23^ To identify a common downstream mediator from all platelet receptors that explains the antiplatelet effect of CCF0054500, we first used an unbiased mass spectrometry approach to interrogate the platelet phosphoproteome. These studies revealed that CCF0054500 regulates proteins involved in cytoskeleton organization, actin filament depolymerization, and regulation of platelet organelles in human platelets (Figure 7A; Supplemental Dataset for Figure 7A, Excel S1). Using a targeted phosphokinase array, we identified HSP (heat shock protein)–27 as the primary substrate phosphorylated after platelet stimulation by CCF0054500, specifically at residues Ser78 and Ser82 (Figures 7B and 7C). This observation was further validated in a different group of human subjects by performing immunoblotting. We observed dose-dependent phosphorylation of HSP27 at residues Ser15, Ser78, and Ser82 by CCF0054500 (875 nM–112 μM) (Figure 7D).

Given the established role of platelet integrin αIIbβ3 (GPIIb/IIIa [Glycoprotein IIb/IIIa]) as a common mediator of platelet activation from the inside to outside signaling and its known role as a mediator of mechanotransduction and platelet shape change,^24,25^ we assessed platelet spreading on a fibrinogen matrix, a process known to depend on the reorganization of the filamentous actin cytoskeleton.^26^ Costaining for platelet filamentous actin (F-actin) and P-selectin revealed that CCF0054500 treatment impaired platelet spreading on the fibrinogen matrix, evidenced by a reduced surface area compared with vehicle (Figure 7E). These findings further highlight the role of CCF0054500 in platelet actin cytoskeleton rearrangement. To better translate platelet mechanotransduction that precedes platelet shape change and subsequent effects on thrombus stability and growth, we evaluated OR2L13-mediated changes in platelet shape and the kinetics of F-actin reorganization. In an ex vivo clot retraction assay using isolated, washed platelets from healthy donors incubated with CCF0054500, we assessed clot retraction—driven by interactions between fibrin and the actin–myosin cytoskeleton and mediated by the mechanosensor integrin αIIbβ3 (GPIIb/IIIa)—which is proportional to the degree of platelet activation and cytoskeletal rearrangement.^24^ Images of washed platelets in the presence of thrombin, with or without CCF0054500, were taken periodically for 60 minutes (Figure 7F). Platelet activation by thrombin caused clear and progressive cytoskeleton rearrangement and clot retraction (Figure 7F, lane 2 versus lane 1), which was inhibited when comparing CCF0054500 treatment with vehicle (lane 4 versus lane 3). To demonstrate the dependence of HSP27 on this biomechanical process, the known HSP27 inhibitor brivudine^26^ was added, showing complete abrogation of the protective effect of CCF0054500 against thrombin-mediated clot retraction (lane 6 versus lane 4). Quantitative analysis of clot retraction (Figure 7G) showed robust and statistically significant differences at all measured time points between vehicle- and CCF0054500-treated platelets, as well as between CCF0054500 and brivudine- plus CCF0054500-treated groups. We further validated the relationship between OR2L13 activation by CCF0054500, downstream phosphorylation of HSP27, and last, actin cytoskeleton rearrangement in platelets that leads to inhibition of platelet reactivity. We immunoprecipitated CCF0054500-activated platelet protein lysate using a HSP27 Ser78 antibody with or without pretreatment with brivudine (a HSP27 inhibitor) (Figure S9). In cluster B, sample 2 (platelets treated with CCF0054500 and immunoprecipitated with the HSP27 Ser78 antibody), there was protein enrichment for complexes involving Rho GTPase (guanosine triphosphatase)–mediated cytoskeleton organization, as well as cell–extracellular matrix interactions, cell-cell communication, and cell junction organization pathways. In contrast, pretreatment with brivudine before CCF0054500 incubation abolished these enrichments, indicating that HSP27 phosphorylation is essential for CCF0054500-driven cytoskeletal signaling (Figure 7H; Supplemental Dataset for Figure 7H, Excel File S2). Last, to better understand the bioenergetics of F-actin depolymerization, we performed an in vitro actin binding assay using washed platelets from healthy subjects treated with CCF0054500 (100 μM). CCF0054500 accelerates the release of F-actin from the platelet membranous fraction into the soluble supernatant fraction (Figure 7I, lane 5, vehicle versus control) without depleting the pool of globular actin (Figure S10, lane 5, vehicle versus control) as shown by SDS-PAGE (sodium dodecyl sulfate-polyacrylamide gel electrophoresis) after Coomassie blue staining. These findings confirm that platelet OR2L13 activation by CCF0054500 promotes F-actin depolymerization through a noncanonical HSP27-dependent pathway, thereby reducing platelet reactivity.

### OR2L13 Agonist Suppresses High Residual Platelet Reactivity in Patients With Vascular Disease

**Figure 8.**
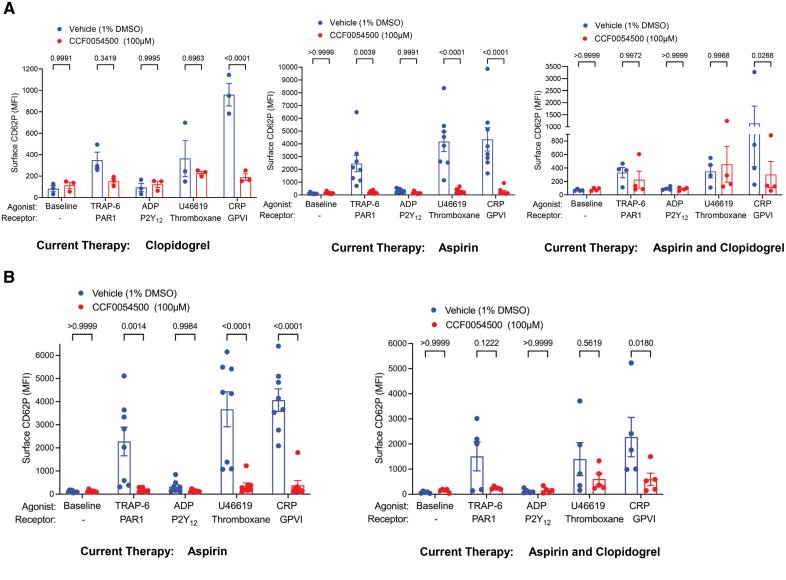
**HRPR mitigated by CCF0054500 in patients with PAD and CAD on antiplatelet therapy. A**, Isolated platelets from patients with atherosclerotic PAD on different antiplatelet treatments (clopidogrel [n=3], aspirin [n=8], and dual antiplatelet therapy with aspirin and clopidogrel [n=4]) were treated with CCF0054500 (100 μM) or vehicle ex vivo for 30 minutes. Platelet reactivity in response to surface receptor agonists was determined (10 μM TRAP-6, 10 μM ADP, 5 μM U46619, and 0.5 μg/mL CRP). HRPR is prominent through the GPVI (glycoprotein VI) receptor in all patients. HRPR is noted through the thromboxane receptor and PAR1 receptor in patients with PAD on aspirin. **B**, Isolated platelets from patients with atherosclerotic CAD on aspirin (n=8) and dual antiplatelet therapy with aspirin, and clopidogrel (n=5) were treated with CCF0054500 (100 μM) or vehicle ex vivo for 30 minutes. Platelet reactivity in response to surface receptor agonists (10 μM TRAP-6, 10 μM ADP, 5 μM U46619, and 0.5 μg/mL CRP) was determined. HRPR is prominent through the GPVI receptor in patients on dual antiplatelet therapy (DAPT). In the patients on aspirin, HRPR is noted through the thromboxane receptor and PAR1 along with GPVI. CCF0054500 suppresses HRPR in these patients. The results are expressed as mean fluorescence intensity of P-selectin ± SEM. *P* values were determined by 2-way ANOVA. CAD indicates coronary artery disease; CRP, collagen-related peptide; GPVI, glycoprotein VI; HRPR, high residual platelet reactivity; OR, olfactory receptor; PAD, peripheral artery disease; PAR1, protease-activated receptor 1; and TRAP-6, thrombin receptor activator peptide 6.

Platelets were isolated from patients with CAD or PAD who were already receiving antiplatelet therapy. Platelets from patients were assessed for their ability to activate through 4 major platelet surface receptors (PAR1, P2Y_12_, thromboxane, and GPVI), with and without exogenous CCF0054500 incubation, after stimulation with TRAP-6 (10 μM), ADP (10 μM), U46619 (5 μM), and CRP (0.5 μg/mL). Patients with both PAD and CAD showed HRPR, most apparent through platelet GPVI, despite dual antiplatelet therapy (aspirin and clopidogrel). In patients with PAD and CAD receiving aspirin, persistent platelet reactivity was also observed through PAR1 and thromboxane receptors along with GPVI. Irrespective of whether the patient was taking mono-antiplatelet therapy (aspirin or clopidogrel alone) or dual antiplatelet therapy (aspirin and clopidogrel together), the addition of CCF0054500 suppressed HRPR through all common signaling pathways of platelets in PAD (Figure 8A) or CAD (Figure 8B). This suggests a noncanonical OR signal transduction pathway in platelets downstream from OR2L13 as a point of common convergence that disrupts platelet activation through the disparate receptor pathways assessed.

We evaluated the off-target effect of CCF0054500 against 78 GPCRs, ion channels, and membrane transporters including assays for Gαs-mediated cAMP production, Gαq-mediated Ca^2^^+^ mobilization, nuclear hormone receptor translocation, and selected ligand-gated K^+^/Na^+^ channels (Eurofins; Figure S11A). Within a dose range of 0.1 to 1000 nM, CCF0054500 demonstrated high specificity, with limited nonspecific effects observed on the phosphodiesterase E2, NAV1.5 sodium channel, cannabinoid receptor 2, and nicotinic acetylcholine receptor D2 (Figure S11B). Off-target effects of CCF0054500 were further evaluated in vivo. Wild-type and *Olfr168*^-/-^ mice were administered CCF0054500 (5 mg/kg per day intraperitoneally) for 3 consecutive days. Platelets were then isolated from blood and stimulated ex vivo. We observed an inhibition of platelet reactivity measured by the common platelet activation receptor GPIIb/IIIa (Jon/A antibody binding) in wild-type platelets from mice treated with CCF0054500 compared with the vehicle-treated group (*P*<0.0001) when platelet activity was induced by thrombin. Whereas CCF0054500 inhibited wild-type mouse platelet reactivity, it did not inhibit platelet activation in *Olfr168*^-/-^ mice (Figure S11C and S11D), suggesting the specificity of CCF0054500 for *Olfr168* (murine ortholog of OR2L13). Although CCF0054500 clearly contributes to some cAMP production, consistent with operating through G_olf_ (G olfactory) and producing cAMP through adenylyl cyclase activation (Figures S1 and S12), the absence of cAMP-mediated PKA (protein kinase A) phosphorylation in platelets by CCF0054500 (Figure 7) and the persistent inhibition of platelet reactivity in response to CCF0054500 in the presence of a PKA inhibitor (not shown) suggested this pathway is of lesser importance than CCF0054500 signaling through HSP27.

## DISCUSSION

This study identified a new class of antiplatelet drugs that operate through the orphan platelet GPCR OR2L13 through a noncanonical pathway. When OR2L13 is activated by CCF0054500, platelet activation is suppressed without an impact on hemostasis or the coagulation cascade. CCF0054500 is equally effective at preventing thrombosis in isolated platelets, platelet‑rich plasma, and whole blood from both humans and mice, suggesting a conserved, cross‑species protective signaling pathway.

Dual antiplatelet therapy, which includes aspirin and a P2Y_12_ receptor antagonist clopidogrel, is prescribed for every patient after acute MI, according to established guidelines.^27,28^ However, some patients are resistant to the P2Y_12_ antagonist clopidogrel.^4,29–32^ We discovered in several patients with CAD and PAD taking antiplatelet medications that HRPR was apparent, and suppression by the addition of CCF0054500 was irrespective of the receptor agonist used to activate platelets.Administration of CCF0054500 to mice revealed it to be fully bioavailable, preventing platelet-mediated arterial and venous thrombosis independent of disruptions in the coagulation cascade. Also, genetic deletion of *Olfr168*, which is the murine OR2L13 ortholog, did not promote bleeding or cause blood loss anemia in a murine model, adverse effects that we see with aspirin. Last, in a murine model of MI, persistent HRPR was noted several days after MI and suppressed by CCF0054500, coincident with improved myocardial function, comparable to mice treated with aspirin—further emphasizing the antiplatelet effect of CCF0054500.

The identification of an additional platelet GPCR target like OR2L13, coupled with a promising chemical probe like CCF0054500, offers an alternative method to circumvent a dysfunctional platelet signaling pathway in patients with clopidogrel resistance, poor response to aspirin therapy, or with recurrent clinical thrombosis despite clopidogrel or aspirin. Prescription antiplatelet medications that include aspirin, P2Y_12_ receptor antagonists, and especially the PAR1 antagonist vorapaxar prevent thrombosis at the expense of impairing the protective response of hemostasis, which is a normal physiological response to injury, and limits excessive bleeding.^23,33^ Because every prescribed antiplatelet drug impairs hemostasis and elevates bleeding risk, clinicians are often compelled to discontinue antiplatelet therapy essential for thrombosis prevention, thus creating significant challenges in patient management. CCF0054500 attenuated all platelet surface receptor pathways that prescription drugs are targeted against, with the notable preference for its marked inhibition of GPVI signaling. GPVI is well-known to be a potent platelet signal transduction pathway for activating thrombosis without interfering with the protective mechanism of hemostasis, and this may explain the mechanism of action of CCF0054500.^34–37^ Preliminary data from murine models in vivo demonstrate that our OR2L13-activating compound CCF0054500 is systemically bioavailable and inhibits thrombosis without impairing hemostasis.^37^ No currently available prescription antiplatelet therapy is capable of achieving this effect. An additional advantage of using a nonodorant OR ligand to target an OR is that it circumvents several chemical limitations inherent to odorant ligands. Odorants are generally unsuitable as therapeutic agents because of their volatility and poor bioavailability, which restrict clinical utility. Moreover, odorants exhibit considerable promiscuity, activating multiple receptors among the approximately 400 ORs in humans, thereby compromising specificity.^38–40^ Although OR2L13 stimulation generates cAMP (Figure S12), a known potent inhibitor of platelets, the noncanonical pathway we discovered to be activated by CCF0054500 operates through the common downstream mediator HSP27. HSP27 appears to be the dominant mechanistic explanation for the antiplatelet effect of CCF0054500 given complete reversal of this protective effect when a HSP27 inhibitor is introduced. HSPs have emerged as promising targets for thrombosis prevention, with HSP47 recently shown to inhibit thrombus formation in immobilized humans and in hibernating bears.^41^ Indeed, HSP27 was shown previously to translocate and to associate with the actin cytoskeleton as a phosphoprotein in activated platelets—a mechanism fully reproduced in our study.^42^ Another biochemical study demonstrated that phosphorylation of HSP on Ser15, Ser78, and Ser82 in platelets impaired F-actin polymerization—a process reversed by mutation of those sites to prevent phosphorylation—although the impact on platelet activation was not assessed.^43^ Our data confirms these previous observations. Platelets adhere to extracellular matrix–coated surfaces, triggering sequential events like shape change, filopodia protrusion, and flattening. Platelets treated with CCF0054500 did not spread on a fibrinogen-coated surface, and CCF0054500 depolymerized actin filaments. Actin polymerization in platelets allows for the formation and elongation of filopodia and lamellipodia protrusions, and likely controls regulated exocytosis of thrombogenic granules, which was an altered process in this study, and provides a mechanistic explanation for the antiplatelet effect of CCF0054500. Filopodia in platelets promote adhesion and limit blood flow, leading to platelet flattening and stable thrombus formation.^44^ Our current data show that when HSP27 is phosphorylated at Ser15, Ser78, and Ser82, it destabilizes and depolymerizes long F-actin filaments, which may limit efficient fusion of prothrombotic granules with the plasma membrane and subsequent exocytosis. This was demonstrated by the significant reduction of P-selectin release coinciding with decreased platelet aggregation. Thus, Ser15 as shown by Butt and colleagues may be a stimulus for F-actin depolymerization.^43^ Yet another study showed phosphorylated HSP27, when released from platelets, promotes activation, so its presence inside platelets may prevent deactivation.^45^ Coimmunoprecipitation of platelets treated with CCF0054500 using a HSP27-Ser78 antibody revealed enrichment of pathways involving Rho GTPases, including activation of IQGAPs (IQ motif-containing GTPase-activating proteins) and formins. Rho GTPases are key regulators of the actin cytoskeleton. IQGAPs bind F-actin through their CH (calponin homology) domain and influence cell shape and motility by modulating the globular actin/F-actin balance. GPVI signaling is known to induce profound rearrangements in the platelet cytoskeleton,^46^ and this may be a reason for observing the antiplatelet effect of CCF0054500 so prominently after platelet activation by CRP.

The persistence of atheroembolism in patients with existing vascular disease remains a problem explained by persistent platelet activation.^47,48^ A significant finding in this study is that HRPR through GPVI is observed in patients with CAD and PAD regardless of the antiplatelet agents prescribed. GPVI is a known mechanosensor for platelet activation.^49^ CCF0054500 prevents thrombosis in blood under high shear conditions of 1,500 s^-1^ but not at 600 s^-1^ in a collagen-coated microfluidics system that promotes shear-mediated platelet activation through GPVI. This is consistent with our mechanistic data that CCF0054500 depolymerizes the actin cytoskeleton and theoretically could make the platelet membrane less susceptible to external biomechanical forces that promote platelet activation at higher shear rates. We previously demonstrated OR2L13 trafficking to healthy platelet membranes under disturbed blood flow conditions^19^ and confirmed this in the present study in a murine MI model, where post-MI platelets exhibited increased surface Olfr168 expression. This mechanism, likely present in patients with CAD and PAD, reveals a unique antiplatelet property of CCF0054500—distinct from existing therapies—highlighting its potential as a first-in-class agent.

Our results indicate CCF0054500 suppresses platelet reactivity as effectively as mono-antiplatelet therapy or dual antiplatelet therapy, and it uniquely does so by interfering with downstream common pathway for platelet activation through all cell surface receptors. Patients who are prescribed clopidogrel, aspirin, or dual antiplatelet therapy often display HRPR, which increases the risk of ischemic vascular events.^29,50^ We show in this study that HRPR in platelets from patients with CAD and PAD is effectively suppressed by CCF0054500, which appears to have few off-target effects on other GPCRs and additional common drug targets like ion channels. When given to mice, CCF0054500 has favorable exposure at low doses, preventing platelet activation and thrombosis in multiple animal models without affecting hemostasis. This makes the development of a platelet OR2L13-modulating ligand a potential innovative alternative for antiplatelet therapy in elderly patients who have a higher incidence of HRPR with both clopidogrel and ticagrelor.^50^

Platelets play a pivotal pathophysiological role in arterial thrombus formation and coronary artery occlusion. It has been noted that platelet reactivity is elevated in the first 6 months after acute ST-segment–elevation MI despite antiplatelet therapy,^51^ and these patients are treated with antiplatelet therapy as a part of secondary prevention. CCF0054500 successfully inhibited platelet reactivity and improved cardiac function in vivo after MI.

We acknowledge limitations of this study including an EC_50_ for CCF0054500 in the low micromolar range. Subtle modifications of the core molecular structure of CCF0054500 are underway to refine the compound and enable traditional drug-like properties. In addition, although we show parenteral delivery of CCF0054500 is an effective antithrombotic medication in mice without affecting hemostasis, oral bioavailability should be prioritized alongside thorough pharmacokinetic studies to better predict the drug behavior in vivo. Although CCF0054500 does not increase bleeding in mice, it is unclear at this time whether the compound will adversely affect hemostasis if introduced into humans.

In conclusion, CCF0054500 is the first nonodorant ligand identified for activating an OR that inhibits thrombosis without impairing hemostasis, by depolymerizing the platelet actin cytoskeleton to limit platelet shape change irrespective of the mechanism used to activate platelets. Targeting platelet OR2L13 in this manner may also have the added advantage of preventing biomechanical platelet activation in pathological conditions such as stenotic atherosclerotic disease, or when platelets are exposed to shear stress by external mechanical cardiac support devices in patients with cardiogenic shock.

## ARTICLE INFORMATION

### Acknowledgments

The 8000 bioactive compound library and resynthesised compounds were provided by the Center for Therapeutics Discovery, Cleveland Clinic Foundation, Cleveland, OH. We are indebted to Dr Anshul Mishra for assistance in molecular cloning.

### Author Contributions

The authors report the following contributions: conceptualization, T.M., J.L., S.S., V.P.V.N.J., and S.J.C.; methodology, A.A., N.W., M.Y., Y.J.S., Q.P.K., R.S., S.G., M.G., M.A., H.E.P., A.R.S., J.G., S.M.S., and S.J.C.; formal analysis, A.A. and S.J.C.; investigation, A.A., N.W., M.Y., Y.J.S., Q.P.K., R.S., S.G., M.G., M.A., H.E.P., A.R.S., J.G., S.M.S., and S.J.C.; patient and control sample collection, C.J., B.R., and A.S.; software analysis, N.S.; resources, S.J.C.; data curation, A.A., N.W., M.Y., Y.J.S., Q.P.K., R.S., S.G., M.G., M.A., H.E.P., A.R.S., J.G., S.M.S., and S.J.C.; writing—original draft, A.A. and S.J.C.; writing—review & editing, A.A, N.W., M.Y., Y.J.S., Q.P.K., R.S., S.G., M.G., M.A., H.E.P., A.R.S., S.M.S., J.G., T.M., J.L., S.S., V.P.V.N.J., and S.J.C.; visualization, A.A.; supervision, S.J.C.; funding acquisition, S.J.C. All authors have read and agreed to the published version of the article.

### Disclosures

S.J.C. reports serving on data and safety monitoring board for Sanofi Inc. within the last 2 years. The other authors report no conflicts.

### Supplemental Material

Supplemental Methods

Figures S1–S12

Videos S1 and S2

Excel Files S1 and S2

References 52–54

## Supplementary Material
