# Supplementary material for: Olfactory Receptor Activation Reduces Platelet Reactivity and Arterial Thrombosis Through Actin Cytoskeleton Remodeling
Source: Circulation. 2026 Apr 7;153(23):1827–44. doi: 10.1161/CIRCULATIONAHA.125.078927 (PMC13236044; doi:10.1161/CIRCULATIONAHA.125.078927)
Supplement: Supplementary file 1 [file cir-153-1827-s001.pdf]

SUPPLEMENTAL MATERIAL

SUPPLEMENTAL METHODS

High Throughput Screen (HTS)

An OR2L13 HEK293 cAMP-Response-Element (CRE) reporter cell line which was previously developed in our lab was used.[19] Stimulation of G_{olf} and adenylyl cyclase by 8,000 bioactive library ligands (Life Chemicals, Inc. Niagara-on-the-Lake, Ontario, Canada) were determined by cyclic AMP (cAMP) production using the cAMP/PKA Signaling Pathway CRE/CREB Reporter cell line (Luciferase) constructed by us and described previously.[19] This HEK293 cell line uses the cAMP Response Element (CRE) to drive a luciferase reporter when cAMP is made, activating PKA which, in turn, phosphorylates CRE binding protein (CREB) and promotes binding to CRE. HEK293 cells have the firefly luciferase gene under the control of multimerized cAMP response element (CRE) stably integrated. These cells were grown in DMEM with Glutamax (Gibco cat. # 10569-010) supplemented with hygromycin B selection.

<u>On the day of the assay:</u> Firstly, using the robotic system (ECHO 550), 150nL of 10mM stock ligands were added to 384 well plates to the final working concentration of 50µM. Then 30K of OR2L13 HEK293 cAMP reporter cells were plated in 30µL phenol free DMEM media in each well. In each sample plate, 16 wells of each, DMSO (vehicle), 500µM (-) Carvone (positive control for OR2L13) and 3µM forskolin which directly activates adenylyl cyclase and produces cAMP (positive control for endogenous cAMP production). Plates were then incubated for 4 hours in a CO_2 incubator at 37°C. After the incubation step was completed, 30µL of Steadylite Luciferase reagent (Perkin Elmer) was added to each well. Luciferase activity was measured using BioTek Cytation 5 plate reader with gain adjusted to autoscale on the reader. All plates passed a Z' >0.5 to indicate a positive hit on the screen. The luminescence for each ligand was calculated as follows: [(Sample-DMSO)/(Forskolin-DMSO)]*100 to normalize with the maximum forskolin activity and to reduce intra- and inter- assay variability between the different plates (it gives us the values as % maximum forskolin). We found 169 hits of 8,000 compounds. All 169 hits above the 25% threshold of Forskolin (positive control) were validated at a concentration of 50 µM in triplicate using the parental HEK293 cAMP cell line, along with OR2L13 HEK293 cAMP cells, to confirm ligand specificity for the OR2L13 receptor. Only 6 of 12 potential compounds were advanced for further study due to the unavailability of remaining 6 compounds from commercial vendors or the initial repository. Therefore, functional validation was performed using the 6 available compounds in isolated platelets, in platelet-rich plasma, in whole blood *ex vivo*, and *in*

vivo. Following counter-screening with the OR2L13 HEK293 cAMP cell line, the top six compounds (CCF0051970, CCF0052249, CCF0053070, CCF0054432, CCF0054500, and CCF0058399) were tested for dose-response at the following concentrations: $1x10^{-4}$ M, $5x10^{-5}$ M, $2.5x10^{-5}$ M,$1.25x10^{-5}$ M, $6.25x10^{-6}$ M, $3.13x10^{-6}$ M, $1.568x10^{-6}$ M, $7.88x10^{-7}$ M, $3.91x10^{-7}$ M, 1.9510^{-7} M with OR2L13 HEK293 cAMP and HEK293 cAMP cell lines (data not shown). To calculate the half maximal effective concentration (EC_{50}) for the top hits, dose response (195nM to 100µM) was performed.

Validation of initial HTS hits using Dynamic Mass Redistribution on OR2L13-HA cAMP/PKA

Cells were grown in DMEM media supplemented with Fetal Bovine Serum (FBS; 10%), and Pen-Strep (1%) in a humidified atmosphere at 37ºC in 5% CO2. Prior to each Dynamic Mass Redistribution (DMR) experiment, 50,000 cells/well were seeded in a 96-well fibronectin-coated Corning Epic microplate in DMEM media (100 µL) and incubated overnight. The following morning, cells were washed with 1X HBSS buffer with HEPES (20 mM; pH 7.4) multiple times and the cells were resuspended in 1X HBSS buffer with HEPES. Next, the DMR plate was briefly centrifuged (100g for 15 seconds at room temperature) to allow cells to settle at the bottom of the plate and then kept at room temperature (RT) for 1 hour. During measurements, basal DMR response was collected for 15 minutes to obtain a baseline defined as the zero point. Then, different concentrations of each test compound of interest or vehicle were used. The DMR signal was read for 60-90 min in a Corning Epic BT system (Corning Epic).

Platelet Light Transmission Aggregometry

To determine the specificity of the identified ligands (CCF0051970, CCF0052249, CCF0053070, CCF0054432, CCF0054500, and CCF0058399) on human platelets, Light Transmission Aggregometry (LTA) was employed. Blood from healthy volunteers (n=7) was collected in sodium-citrate tubes, as described by us previously, after obtaining informed consent. Within 30–60 minutes of collection, anticoagulated whole blood was centrifuged at 200g for 15 minutes at room temperature (RT) to obtain platelet-rich plasma (PRP). Platelet-depleted plasma (PDP) was obtained from the remaining sample by re-centrifugation at 2,500g for 10 minutes and was used to initiate baseline optical density. Platelet count in each sample was adjusted to 200 million cells per mL in PRP with PDP. PRP from healthy subjects was pre-treated with 100 µM of identified ligands (CCF0051970, CCF0052249, CCF0053070, CCF0054432, CCF0054500, and CCF0058399) for 30 minutes at 37°C. Platelet aggregation was measured following stimulation with platelet agonist adenosine diphosphate (ADP) (1–5 µM), where formation of platelet

aggregates leads to increased light transmission. ADP was used as the initial screen in a specific assay for platelet reactivity (LTA) given that it operates through the P2Y12 receptor, a G-protein-coupled receptor (GPCR) operating through Gαi, which inhibits adenylyl cyclase and activates platelets through decreased cAMP production. This directly opposes the effects of OR2L13 ligands that signal through Gα(olf), which activates adenylyl cyclase and inhibits platelets in part through endogenous cAMP production. For data analysis, % aggregation in response to ADP was compared between ligands and vehicle (DMSO).

Platelet Flow-Cytometry

Ligands (CCF0054500, CCF0052249 and CCF0058399) from the initial HTS were assessed for their platelet inhibitory effect by assaying surface P-Selectin expression (CD62P) through platelet receptors as a surrogate for α-granule exocytosis. Platelets were isolated from whole blood collected from healthy volunteers (n=10) in sodium-citrate tubes. Whole blood was centrifuged at 200g for 15 minutes at RT, and PRP was collected. Collected PRP was centrifuged again at 200g for 10 minutes to remove remaining WBCs and RBCs. Then PRP was collected in clean Falcon tube and was resuspended in Tyrode's buffer at 1:1 ratio with the addition of Prostaglandin I2 (PGI_2, 10nM) and was centrifuged at 1,400g for 5 minutes to get platelet pellet. Washed platelets were resuspended in 1mL of Tyrode's buffer and pre-treated with ligands (CCF0054500, CCF0052249 and CCF0058399) or vehicle (DMSO) for 30 mins. Then, 4mL of Tyrode's buffer was added to adjust the platelet count to 50 million cells per mL. Washed platelets were finally stimulated with the PAR1 agonist TRAP-6 (10μM), the thromboxane receptor agonist U46619 (5μM), the $P2Y_{12}$ receptor agonist ADP (10μM) or the GPVI receptor agonist CRP (0.5μg/mL) for 15 minutes. Platelets were then incubated with phycoerythrin (PE)-conjugated anti-CD62P for 30 minutes at RT prior to fixation with 2% formalin. Samples were analyzed using flow cytometry (BD Accuri C6 Plus) to acquire 10,000 events. Data was analyzed using FlowJo software.

Ex-vivo Thrombosis Model (biomechanical platelet activation)

Platelets are activated biomechanically by shear stress in atherosclerotic and aneurysmal arteries. To mimic these pathological processes, the Total Thrombus formation Analysis System (T-TAS01, DiaPharma) microfluidics system was utilized to determine the effect of lead OR2L13 ligand on thrombosis in whole blood. As whole blood travels through collagen-coated capillaries under high shear (1,500 s-1) or low shear (600 s-1), platelets adhere to the collagen surface and release ADP and Thromboxane A_2, which amplifies activation. This leads to the formation and growth of the platelet thrombus, and channel occlusion that is determined by increasing pressure

as a function of time. The effect of 100 μM CCF0054500 (lead compound) was tested in the whole blood from healthy subjects (n=7) using PL (shear stress 1,500 s^{-1}) and AR chips (shear stress 600 s^{-1}).

In vivo **arterial thrombosis model by laser injury**

Thrombus formation in response to laser injury was measured in real-time as previously described.[52-54,] FVB/NJ male mice, aged 8 weeks were utilized for their known high expression of platelet *olfr168* (purchased from The Jackson Laboratory). Mice were treated with a daily dose of 75% (w/v) DMSO vehicle control or 5mg/kg per day CCF0054500 intraperitoneally for three consecutive days. Ninety minutes after the third treatment, mice were anesthetized with an intraperitoneal injection of 125 mg/kg ketamine and 12.5 mg/kg xylazine. A patch of skin was removed from the neck, and the jugular vein was cannulated with polyethylene tubing. Mice were intubated with polyethylene tubing for use with a respirator. The cremaster muscle was exteriorized and pinned onto a custom intravital microscopy tray. Platelet and fibrin accumulation were measured by infusing Dylight 488-labeled anti-platelet antibody (CD42b; 0.1 mg/kg body weight; Emfret Analytics) or Dylight 647-labeled anti-fibrin (clone 59D8; 0.3 mg/kg) monoclonal antibody through a jugular vein catheter. The cremaster muscle arterioles were injured using a MicroPoint Laser system (Andor, Belfast, UK). Data was acquired before and after laser injury using the brightfield, 488/520 nm, and 640/670 nm channel. Images were captured for 250 seconds at 2 frames/second using a CCD camera (ORCA Flash 4.0, Hamamatsu Photonics, Japan). Data was analyzed using Slidebook 6.0 (Intelligent Imaging Innovations, CO). Data from 29-31 thrombi were used to determine the median value of the integrated fluorescence intensity to account for variability in thrombus formation at any given experimental conditions. The integrated fluorescence intensity (RFU) for platelets and fibrin accumulation was calculated for each frame per the following equation:

$$
\begin{aligned}
&Integrated\ fluorescence\ intensity \\
&\quad = Sum\ Intensity\ of\ the\ signal \\
&\quad - \ (Average\ of\ the\ maximal\ background\ intensity \\
&\quad \times\ Area\ in\ pixel\ of\ the\ signal)
\end{aligned}
$$

The area under the curve (AUC) was calculated for individual thrombi using the trapezoid method with Igor Pro 9 (WaveMetrics, Inc., OR) and normalized to injury lengths to evaluate statistical significance. Injury lengths (μm) were determined in the brightfield channel as the distorted region along the luminal face of the vessel following the injury.

IVC constriction thrombosis model

FVB/NJ male mice were obtained from The Jackson Laboratory and housed on a standard 12 h dark/light cycle. Mice were treated with a daily dose of 75% (w/v) DMSO vehicle control or 5 mg/kg per day CCF0054500 intraperitoneally for three consecutive days. Ninety minutes after the third treatment, mice were anesthetized by continuous isoflurane induction (2%), with 100% oxygen at a 2 L/min rate and placed on a heated pad at 37°C. The abdomen was incised, and intestines were exteriorized to allow visualization of the inferior vena cava (IVC). All visible side branches of the IVC were ligated with 7-0 PROLENE sutures (Ethicon, Norderstedt, Germany) while back branches from the renal veins to the iliac bifurcation were cauterized. The IVC was then separated from the aorta and ligated in the same manner as the IVC side branches. The abdominal cavity was closed using 5-0 vicryl sutures (Ethicon) and adhesive skin glue. Mice were sacrificed 48 hours later and IVC thrombi were harvested, weighed, fixed by incubation in 4% (v/v) paraformaldehyde overnight at 4°C, and embedded in paraffin.

Tail bleeding assay

Tail bleeding assay was performed with FVB/NJ wild-type, heterozygous and homozygous-deficient Olfr168$^{-/-}$ mice (murine ortholog of human OR2L13). Animals were bred in-house. Mice were treated with a daily dose of 5mg/kg per day CCF0054500 IP for three consecutive days. Another group of FVB/NJ male mice was treated with CD42b (GPIbα) antibody (0.5µg/g once IP) from Emfrets R300 antibody. Following the treatment, mice were anesthetized with an IP injection of 90 mg/kg ketamine and 10 mg/kg xylazine. A distal 3-mm segment of the tail was amputated with a scalpel followed by the tail being immersed in a pre-warmed isotonic saline solution in the 50-mL Falcon tube. Each animal was monitored for 9 minutes even if bleeding stopped, to detect any re-bleeding. Time to cessation of bleeding was evaluated in minutes followed by animal sacrifice with Ketamine and xylazine.

Platelet Flow-Cytometry

Ligands from the initial HTS were assessed for their platelet inhibitory effect by assaying surface P-Selectin expression (CD62P) through additional platelet receptors as a surrogate for α-granule exocytosis. Platelets were isolated from whole blood collected from healthy controls in sodium-citrate tubes. Whole blood was centrifuged at 200g for 15 minutes at RT and PRP was collected. Collected PRP was centrifuged again at 200g for 10 minutes to remove remaining WBCs and RBCs. Platelets were washed using Tyrode's buffer at a 1:1 ratio with the addition of Prostaglandin I2 (PGI_2; 10 nM) and the platelet count was adjusted to 50 million per mL with Tyrode's buffer.

Then these washed platelets were resuspended in 1 mL of Tyrode's buffer. The washed platelets were pre-treated with ligands and vehicle (DMSO) for 30 mins. Then, 4 mL of Tyrode's buffer was added to make up the volume. After that, washed platelets were stimulated with the PAR1 agonist TRAP-6 (10μM), a thromboxane receptor agonist U46619 (5μM), a $P2Y_{12}$ receptor agonist ADP (10μM) and a GPVI receptor agonist CRP (0.5μg/mL) for 15 minutes. Platelets were then incubated with phycoerythrin (PE)-conjugated anti-CD62P for 30 minutes at RT prior to fixation with 2% formalin. Samples were analyzed using flow cytometry (BD Accuri C6 Plus) to acquire 10,000 events. Data was analyzed using FlowJo software.

Confocal Microscopy

Glass coverslips were coated with 200 μg/mL human fibrinogen in 1X PBS followed by aspiration and drying in a sterile hood for 30 minutes. Washed platelets from healthy controls were isolated and resuspended in 1 mL fresh Tyrode's solution. Washed platelets were then incubated with 100μM CCF0054500 and vehicle (DMSO) at 37°C for 30 minutes, then transferred to the coverslip and placed in an incubator at 37 °C for 30 minutes. Excess (non-adherent) platelets were washed with 1X PBS, followed by the addition of 10% formalin fixation for 20 minutes at RT. The coverslip was then washed with 1X PBS three times. Then 0.1% Triton X-100 was added to the plate and was incubated for 30 minutes at RT. Again, the plate was washed with 1X PBS for three times. P-Selectin (Alexa Fluor ® 546 sc-8419 from Santa Cruz) and Rhodamine Phalloidin (3 μL) diluted in PBS were added to the plate and placed in the dark at RT for 60 minutes. Excess antibody was washed with 1X PBS solution, and a drop of Diamond mount was added before imaging. The surface area of platelets ($μm^2$) was calculated using a calibrated signal from random fields of view using an Olympus IX70 Fluorescence Microscope and a 60X oil-immersion lens.

Phosphoproteomics

Phosphokinase analysis: Unbiased phospho-proteomics deciphered the signaling pathway of OR2L13 ligands following lead compound incubation with platelets. Washed platelets from healthy subjects (n=3) were pre-treated with Vehicle (DMSO) or CCF0054500 (100μM) for 30 minutes at 37°C. Treated samples were processed and assessed by unbiased phosphoprotein analysis using liquid chromatography/mass spectrometry ([LC-MS]).

The samples were homogenized in Urea Lysis Buffer (20mM HEPES, 9M urea, 1X HALT protease/phosphatase inhibitor, pH 8.0) with a point sonicator. Protein concentrations were measured using the Pierce BCA kit and a 1.35 mg aliquot of protein was used for in-solution digestion. The samples were reduced by dithiothreitol and alkylated by iodoacetamide. A 25 μg of

sequencing grade trypsin was added to each sample and incubated at RT overnight. The digested samples were desalted using Sep-Pak® C18 1 cc Vac Cartridge (Waters Corporation # WAT023590). These samples were split into two aliquots of 650 µg for FeNTA and TiO2 phosphopeptides enrichment. The 650 µg aliquots were lyophilized using a Labconco FreeZone 6 Liter -84°C Console Freeze Dryer and spiked with phosphopeptides standard (Protea Biosciences Group, Inc. #PS-180-1) prior to phosphopeptides enrichment. One aliquot of each sample was enriched using Hi-Select FeNTA Phosphopeptide Enrichment Kit (ThermoScientific A32992) and the second aliquot was enriched using Thermo Scientific™ High-Select™ TiO2 Phosphopeptide Enrichment Kit (Thermo Scientific # A32993). The eluted peptides were dried immediately in SpeedVac. Each sample was reconstituted in 25 µL 0.1% formic acid and ready for LCMS analysis. The LC-MS system was a ThermoScientific Fusion Lumos mass spectrometry system. The HPLC column was a Dionex 15 cm x 75 µm id Acclaim Pepmap C18, 2µm, 100 Å reversed- phase capillary chromatography column. 5 µL volumes of the extract were injected and the peptides eluted from the column by an acetonitrile/0.1% formic acid gradient at a flow rate of 0.25 µL/min were introduced into the source of the mass spectrometer on-line. The microelectrospray ion source was operated at 1.9 kV. The digest was analyzed using the data dependent multitask capability of the instrument acquiring full scan mass spectra to determine peptide molecular weights and product ion spectra to determine amino acid sequence in successive instrument scans. Some additional instrumental parameters are given below:

LC-MS Method	Instrument	Fusion Lumos<br>Dionex Ultimate 3000 RSLCnano
	Method	2hr-Gradient_CID
	Trapping Column	Acclaim Pepmap C18 100µm x 2cm, 5µm, 100Å
	Analytical Column	Acclaim Pepmap C18 75µm x 25cm, 2µm, 100Å
	Solvent A	0.1 Formic Acid in H2O
	Solvent B	0.1 Formic Acid in Acetonitrile
	Gradient	Time (%B)<br>0 (2%)<br>5 (2%)<br>110 (35%)<br>120 (90%)

		127 (90%)<br>129 (2%)<br>140 (2%)
	MS1 Resolution	120000
	MS1 Range	350-1500 Da
	MS1 AGC	4.0e5
	MS2 Method	CID, Ion Trap Detection
	MS2 Isolation Window	1.6 Da
	MS2 Collision Energy	35%
	MS2 AGC	2.0e3
	DDA settings	3 second duty cycle
	Dynamic Exclusion	1 repeat<br>10 ppm window<br>60 s exclusion

The data were analyzed by using all CID spectra collected in the experiment to search for the mouse SwissProtKB database using the program Sequest bundled into PD2.5.

Search Parameters	Program: Protein ID	Proteome Discoverer v2.5
	Database	Human Swiss ProtKB Downloaded on 3-23-2022 Entries 26576
	Protease	Trypsin, full
	Missed Cleavages	3
	Mass Accuracy	10 ppm MS1<br>0.6 Da MS2
	Variable Modifications	Oxidation of Met<br>N-terminal Acetyl: Protein<br>Phosphorylation S,T,Y
	PTM site localization	ptmRS node
	Static Modifications	Carbamidomethylation of Cis

	Program: Protein/Peptide validation	Percolator
	Protein ID Requirements	1 Peptides
	FDR Rate: Peptide	Peptide Prophet 1%
	FDR Rate: Protein	Peptide Prophet 1%
	LFQ Quantitation	
	Alignment	Minora Feature Detector node
	Normalization	Total Peptide
	Peptides	All
	Imputation	Impute using a normal distribution (Perseus)
	Matrix Reduction	Remove proteins with less than 3 valid values in at least one group (Perseus)

The overall abundance of peptides was moderate with over 16,000 peptides identified, 33% of which were phosphorylated. The phospho-peptides identified in these samples were compared and three filters were used to identify differentially expressed phospho-peptides with a P-value > 0.05.

Targeted phosphokinase array

The results were validated using the Proteome Profiler Human Phospho-Kinase Array Kit (R&D Systems). For the phosphokinase array, washed platelets from healthy controls were treated with 100µM CCF0054500 or vehicle (DMSO) for 30 minutes at 37°C. Platelets were then lysed with lysis buffer and the assay was performed according to the manufacturer's recommendations.

Western blotting

Washed platelet pellets were collected into Laemmli buffer, and proteins were separated by SDS-PAGE on commercially available gradient gels (4-20%, Invitrogen) at 200V at RT. Separated proteins were then transferred to PVDF membranes (Bio-Rad) at 100 V for 1 hour with an ice pack at RT. These membranes were blocked using 3% bovine serum albumin/Tris-buffered saline–Tween 20 buffer for 60 minutes at RT with agitation. Primary antibody was prepared in TBST-T solution (1:1,000 dilution in 3% bovine serum albumin/Tris buffered saline–Tween 20) and incubated for 12 hours at 4ºC with agitation. Secondary antibody (GE Healthcare,

Buckinghamshire, UK) was used in a 1:2,000 titer in 3% bovine serum albumin /Tris-buffered saline Tween 20 for 1 hour at RT with agitation. Imaging was done using the ChemiDoc™ MP Imaging System (Bio-Rad) and blots were quantified by densitometry using ImageJ software (National Institutes of Health).

Co-Immunoprecipitation assay with HSP27-Ser78 antibody

To confirm the findings of phosphoproteomics, co-immunoprecipitation assay was performed with HSP27-Ser78 antibody under following conditions: Platelets treated with vehicle (DMSO), platelets treated with CCF0054500 (100µM), platelets treated with BVDU (60µM) and CCF0054500 (100µM). Protein Extraction: 100 µL of 8M urea was added to the sample, placed it in an ultrasonic cleaner at low temperature for 40 minutes, and then centrifuged to collect the supernatant. SDS-PAGE: 2 µL of the sample was mixed with 2.5 µL of 5× loading buffer and the volume was made up to 10 µL. The sample was heated in boiling water for 5 minutes. 5 µL of the marker and 12.5 µL of the sample were loaded in the wells of the SDS-PAGE gel, then covered with the lid and connected to the electrodes. The voltage was set to 80 V for 15 minutes, then changed to 120 V for 60 minutes. Protein staining: The gel was removed from the plate, washed with double- distilled water to remove SDS and placed in Coomassie Brilliant Blue staining solution at room temperature for 30 minutes. For destaining, the solution was replaced with distilled water, and the gel was placed on a shaker set at 60 rpm, the distilled water was changed every 20 minutes until the gel was completely de-stained.

CO-IP (Co-immunoprecipitation): 100 µL of Protein A beads were equilibrated using 0.5 mL of binding buffer. The process was repeated twice. 5 µL of antibody (HSP27-Ser78) was added to the equilibrated beads and incubated at 4°C for 2-4 hours. The supernatant was discarded. 0.5 mL of washing buffer was added to remove non-specific proteins. The supernatant was discarded, and the washing was repeated three times. 50 µL of the protein sample was added to the equilibrated beads and incubated overnight at 4°C. The supernatant was discarded, and 0.5 mL of washing buffer was added to remove non-specific proteins. The supernatant was discarded, and washing was repeated three times. SDS-PAGE (Post Co- immunoprecipitation): 10 µL of the CO-IP sample was mixed with 2.5 µL of 5× loading buffer, then heated in boiling water for 5 minutes. 5 µL of the marker and 12.5 µL of the sample were loaded into the wells of the SDS-PAGE gel. The voltage was set to 80 V for 15 minutes, then the voltage was changed to 120 V for 60 minutes. Silver Staining: The gel was removed from the plate, and silver nitrate staining was performed according to the kit instructions.

Clot retraction Ex vivo

Washed platelets ($2x10^8$ cells/mL) from healthy controls (with or without preincubation with 60µM BVDU (Brivudine) for 45 minutes at 37°C) were incubated with CCF0054500 or vehicle (DMSO) for 30 minutes at 37°C. Fibrinogen was added at a final concentration of 1 mg/mL with calcium chloride at a final concentration of 2 mM. Finally, Thrombin (1U/mL) was added and then the tubes were incubated at 37°C. Images were captured every 10 minutes for 1 hour and data were presented as clot area.

Coronary artery ligation

Myocardial infarction (MI) was induced by left anterior descending (LAD) coronary artery ligation. To this end, animals received buprenorphine SR (3.25 mg/kg SQ) and were then anesthetized with 2-3% isoflurane inhalation in an induction chamber. Anesthetized mice were placed in a supine position and intubated with a blunt 20-gauge cannula through the larynx and ventilated with a volume cycled rodent ventilator (Harvard Apparatus) with 2-3% isoflurane at a volume of 200-300µL and a respiratory rate of 150/minute. The left thorax was opened in the fourth intercostal space, with all muscles overlying the intercostal space dissected free and retracted. After opening the pericardium, myocardial ischemia was induced by placing a 6- 0 silk suture around the left anterior descending coronary artery (LAD), approximately 2 to 3 mm from its origin. The ligation was deemed successful when the anterior wall of the LV turned pale. The thorax was closed with 6-0 silk, and the skin was sutured using 5-0 polypropylene suture. Animals recovered on a separate heating pad. Sham-operated animals were subjected to the same surgery, except no ligature was placed around the LAD. Echocardiography was performed on these mice to assess the function of the left ventricle (LV) using the Vevo 2100 Imaging System (VisualSonics).

Off-target evaluation of lead compounds on other established drug targets

Reporter cell lines expressing GPCRs and ion channels were utilized to determine off-target effects of OR2L13 ligands (Eurofins DiscoverX, San Diego CA). In brief, the assay read out determines whether putative OR2L13 ligands were agonists or antagonists for more than 70 targets using cAMP production, Ca^{2+} mobilization, ion flux, and nuclear receptor activity readouts. The readout provides potency (IC_{50}, EC_{50}) and efficacy (E_{max}) from a multipoint concentration response-curve (CRC), the top concentration starting at 10 mM.

Thrombosis Assay in Platelet Poor Plasma: Procoagulant activity involving proteins in the coagulation cascade in the absence of platelets and the impact of CCF0054500 were assessed

using the Thrombodynamics ® system (DiaPharma). A volume of 120 µl of platelet poor plasma (PPP) from control subjects was supplemented with vehicle (DMSO) or CCF0054500 and transferred to an Eppendorf tube containing corn trypsin inhibitor (CTI), incubated for 3 min at 37° and then transferred to an Eppendorf tube containing Calcium acetate. Recalcified plasma was placed into the chamber. An insert with immobilized tissue factor was immersed into plasma. Clot growth began from the tissue factor-covered surface. Clot growth was monitored by light scattering using a digital camera for over 60 minutes.

Table 1: Antibodies and key reagents used

Agonist/Antibody	Catalogue Number	Antibody titers	Reference
TRAP-6	3497/5		TOCRIS
2-Methylthioadenosine diphosphate trisodium salt (ADP)	1624		TOCRIS
U46619	1932		TOCRIS
CRP-A			Pplus Products
Thrombin	13188		Cayman Chemical
CD62P Rat anti-Mouse, FITC, Clone: RB40.34, BD	BDB553744	1:100	Thermofisher
CD62P Monoclonal Antibody (AK-4), PE, eBioscience™	12-0628-42	1:100	Thermofisher
Jon/A (PE-labeled Rat Anti-Mouse Integrin αIIbβ3 (GPIIb/IIIa, CD41/61)	M023-2	1:100	Emfret Analytics GmbH & Co. KG
Anti-Tubulin (Mouse IgG1)	3873S	1:8000	Cell Signaling
Anti-GAPDH (Rabbit IgG1)	5174S	1:8000	Cell Signaling

Phospho-HSP27 (Ser82) (D1H2F6) XP® Rabbit mAb	9709T	1:2000	Cell Signaling
Phospho-HSP27 (Ser78) Antibody	2405	1:2000	Cell Signaling
Phospho-HSP27 (Ser15) Antibody	2404T	1:2000	Cell Signaling
HSP27 (G31) Mouse Monoclonal Antibody	2402	1:8000	Cell Signaling
Actin Binding Protein Spin-Down Assay Biochem Kit: human platelet actin	BK013		Cytoskeleton
CD42b antibody for mouse	R300	0.5μg/g	Emfret Analytics

Supplemental Figures

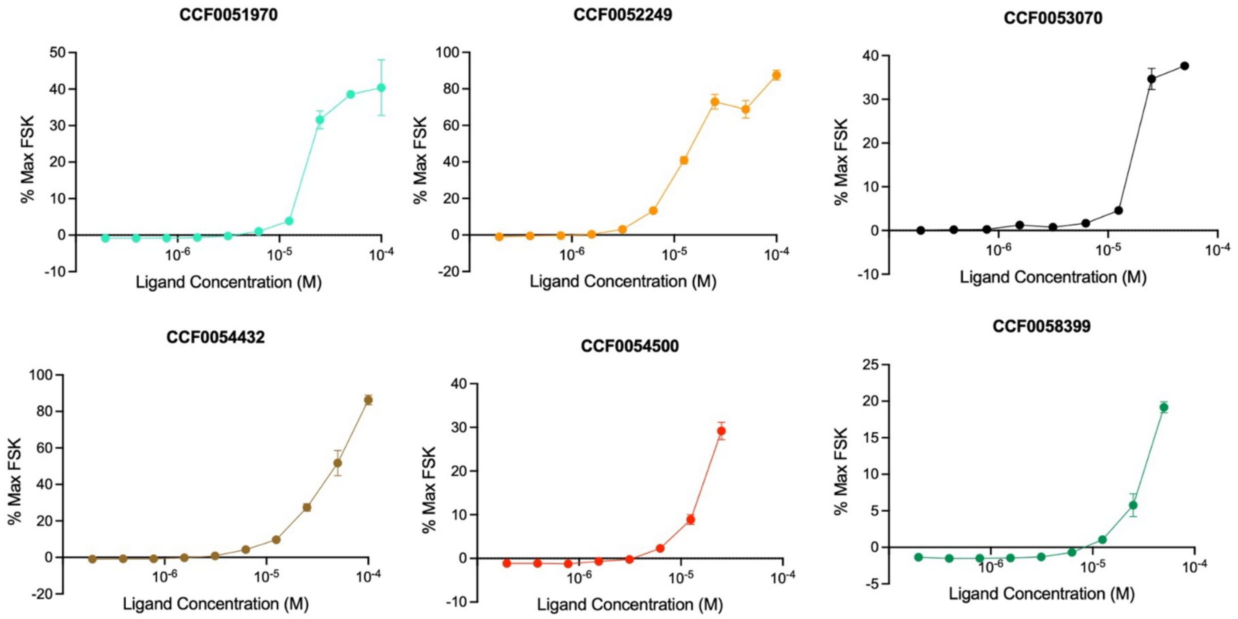

Figure S1: Dose response of hits with 1.5-fold change above baseline (CCF0051970, CCF0052249, CCF0053070, CCF0054432, CCF0054500 and CCF0058399) was determined at different concentrations of ligand in OR2L13 HEK293 cAMP cell line. Different concentrations of ligands used were: $1x10^{-4}$ M, $5x10^{-5}$ M, $2.5x10^{-5}$ M,$1.25x10^{-5}$ M, $6.25x10^{-6}$ M, $3.13x10^{-6}$ M, $1.568x10^{-6}$ M, $7.88x10^{-7}$ M, $3.91x10^{-7}$ M, $1.95x10^{-7}$ M

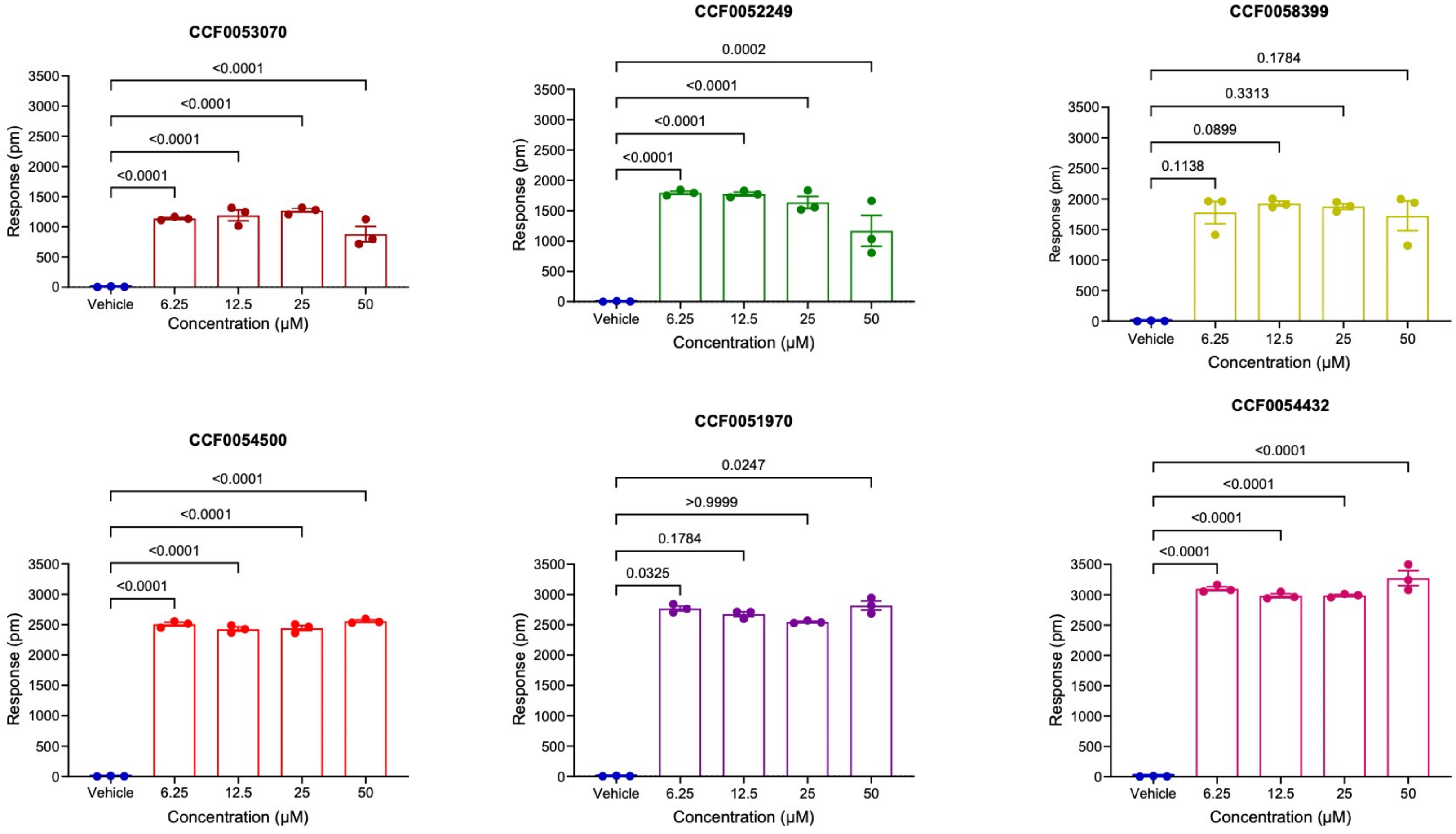

Figure S2: Validation of hits using Dynamic Mass Redistribution (DMR). The effect of top hits, CCF0053070, CCF0052249, CCF0058399, CCF0054500, CCF0051970 and CCF0054432 at different doses (6.25μM, 12.5μM, 25μM and 50μM) was validated using DMR to determine the effect of these ligands on the cellular responses in OR2L13 HEK293 cAMP reporter cell line. All these ligands showed higher cellular responses in comparison to vehicle control (DMSO). *P*- value was determined using One-way ANOVA

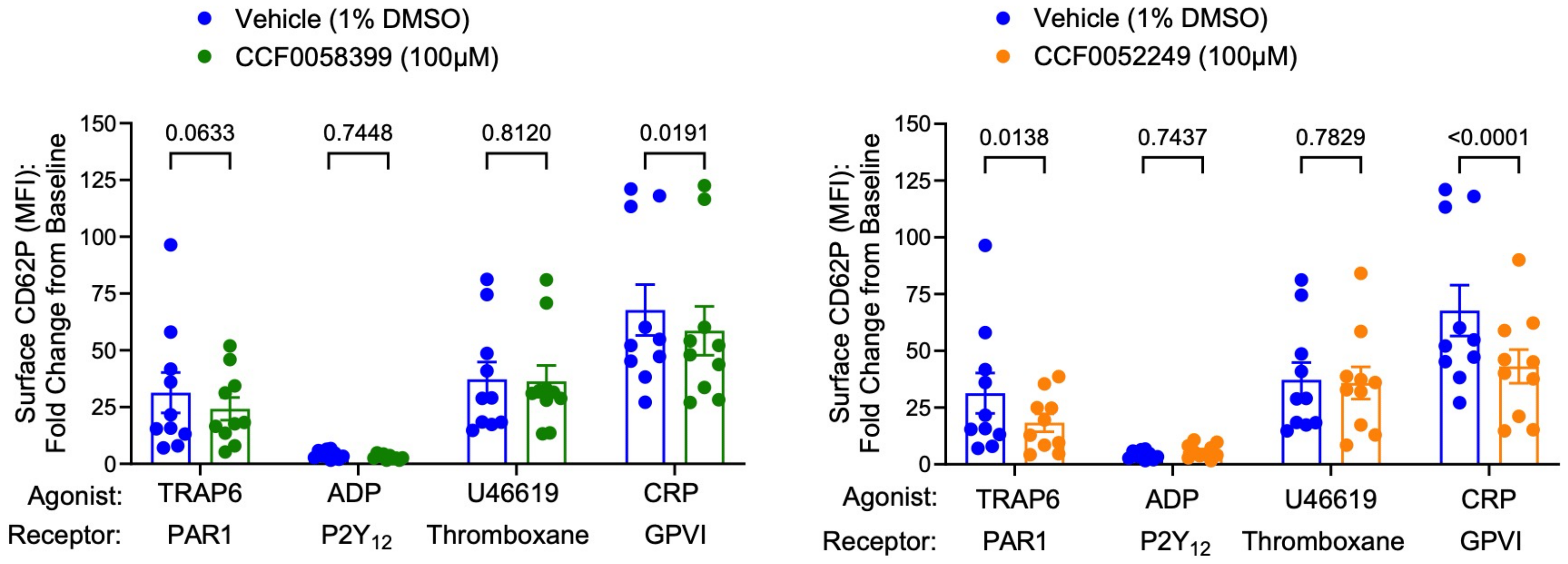

Figure S3: Impact of non-olfactory OR2L13 agonists on Platelet Activation. Platelet activation by alpha granule exocytosis and changes in surface P-selectin expression of washed platelets treated with OR2L13 agonists (CCF0058399 and CCF0052249) and vehicle for 30 minutes at 37ºC. The results are expressed as Mean Fluorescence of P-selectin ± SEM (n=10, t-test). TRAP6=Thrombin Receptor Activator Peptide 6, ADP=Adenosine Diphosphate, CRP=Collagen-Related Peptide.

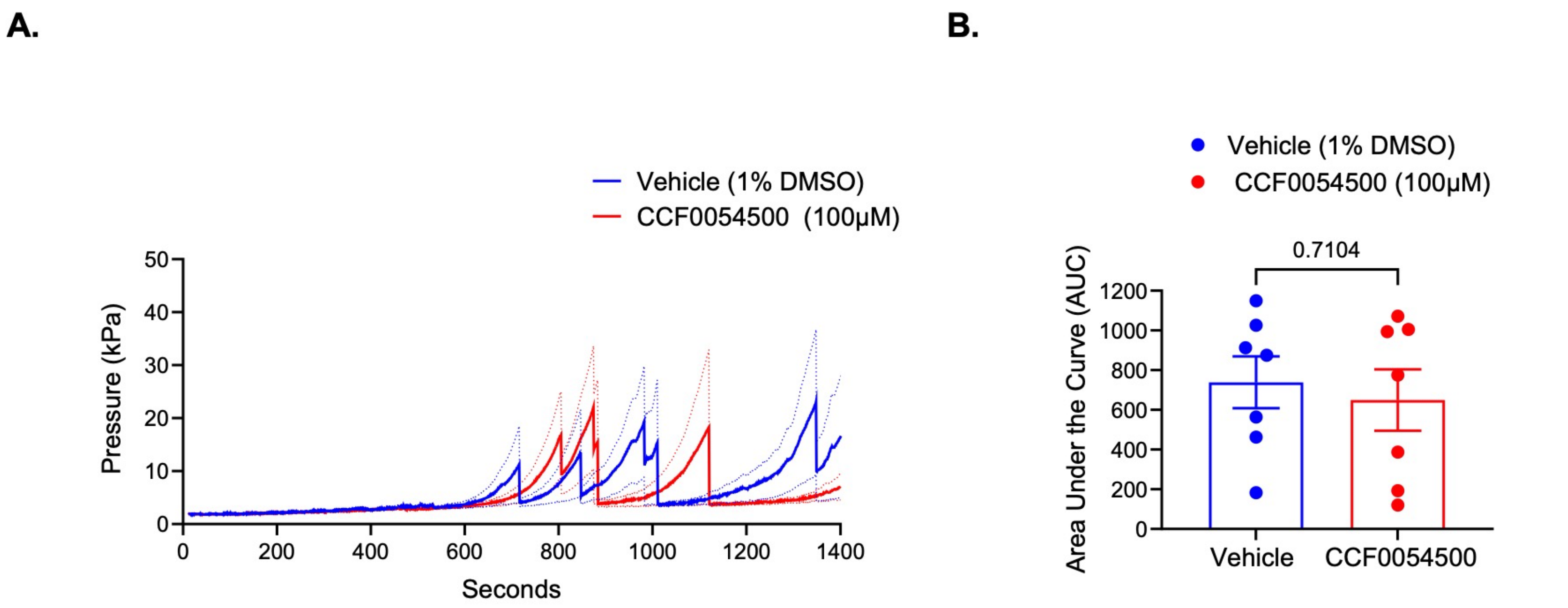

Figure S4: Impact of non-olfactory OR2L13 agonist (CCF0054500) on biomechanical platelet activation under low arterial stress. (A). T-TAS® (Total Thrombus-formation Analysis System) evaluates thrombosis of whole blood under lower arterial shear stress conditions (600 Sec^{-1}) as a function of time and peak pressure. CCF0054500 does not change occlusion by microfluidics under low shear stress (n=7 healthy volunteers). **(B).** Summary data represented as area Under the Curve. Data is represented as mean ± SEM, Mann-Whitney test.

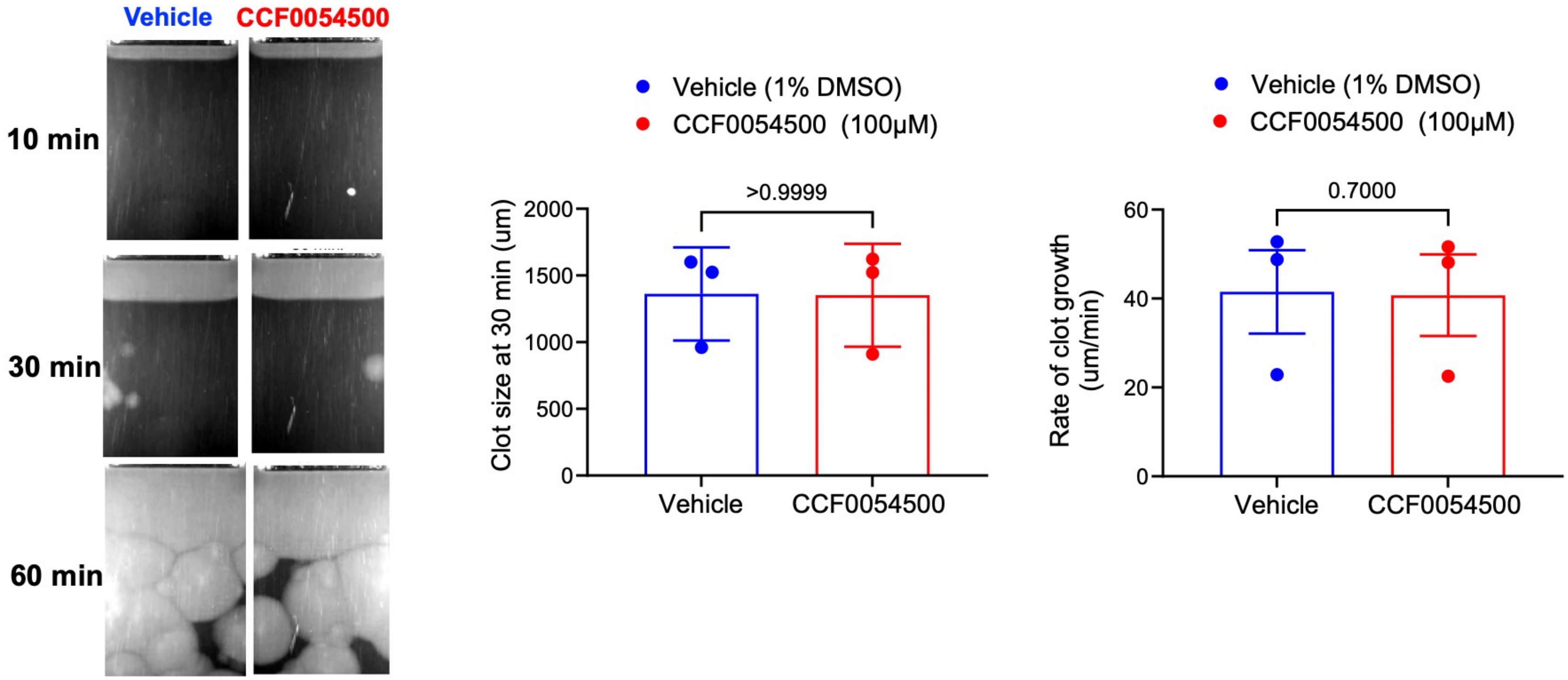

Figure S5: No effect of CCF0054500 on the coagulation cascade. CCF0054500 effect on coagulation was tested through Thrombodynamics ® analysis of Platelet Poor Plasma (PPP). There is no difference in clot size or rate of growth by fibrin formation when treated with CCF0054500 (100μM) compared with Vehicle (DMSO) (n=3). *P* value is measured using the Mann-Whitney *U* test.

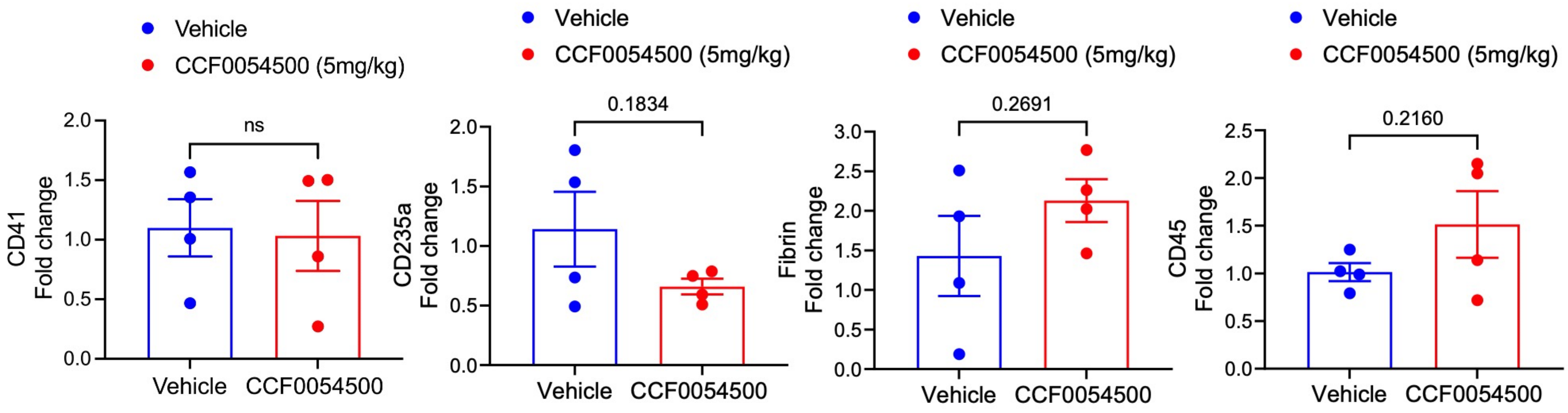

Figure S6: CCF0054500 does not change the cell composition in thrombus in IVC stasis model. RNA was isolated from the isolated thrombus from the different mice groups treated with CCF0054500 (5mg/kg per day) and vehicle. qPCR was performed to assess the different cell populations: Platelets (CD41), Erythrocytes (CD235a), Leukocytes (CD45) and fibrin. No difference was found in the cell populations tested in these two groups. Data is presented as mean ± SEM, n=4 thrombi (t-test).

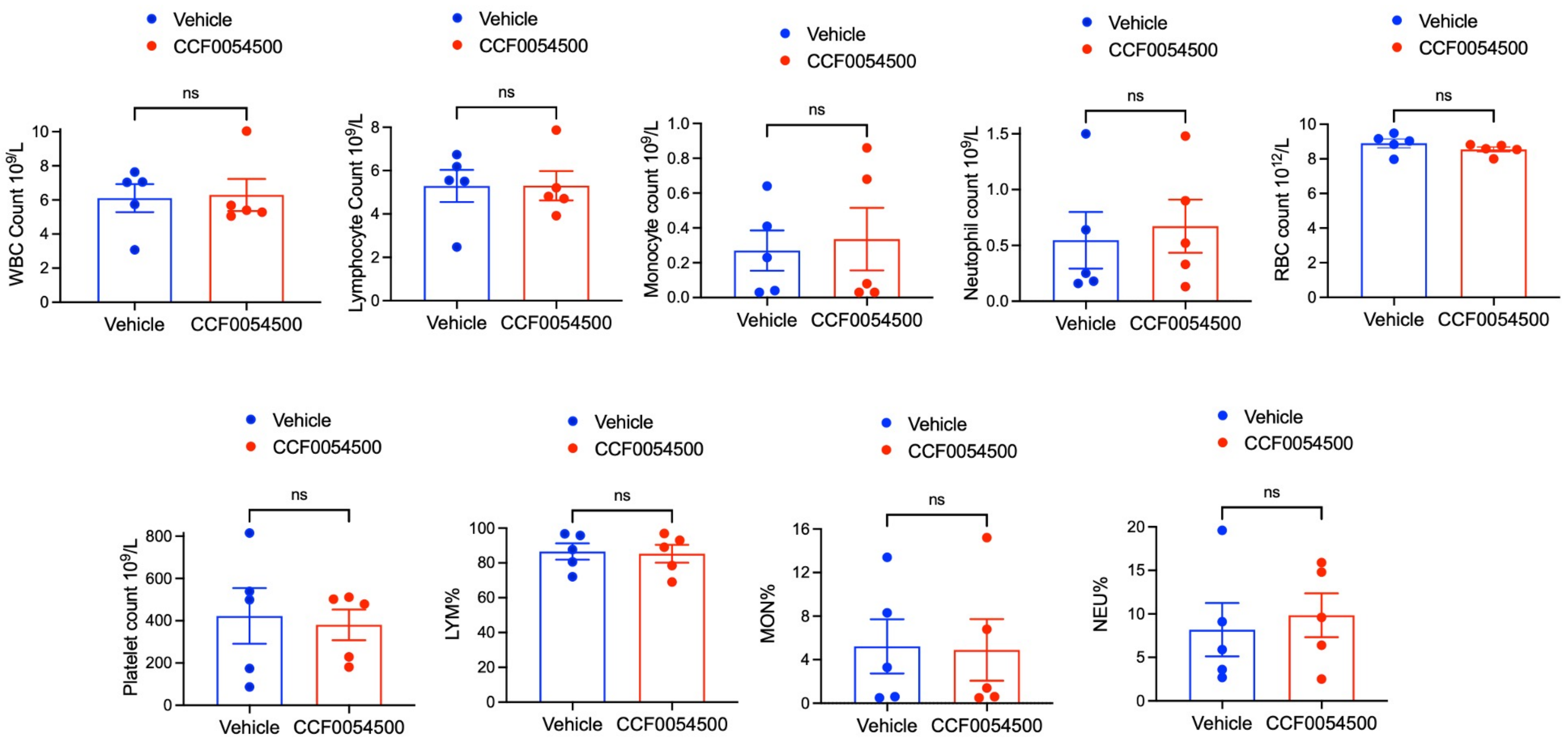

Figure S7: Complete blood count of mice: CCF0054500 does not change circulating cells in the blood of mice. No effect of CCF0054500 (5mg/kg per day) on thrombopoiesis, leukopoiesis, or hematopoiesis. Data is presented as mean ± SEM, n=5 mice in each group (Mann-Whitney U test). RBC=red blood cells. WBC=white blood cells.

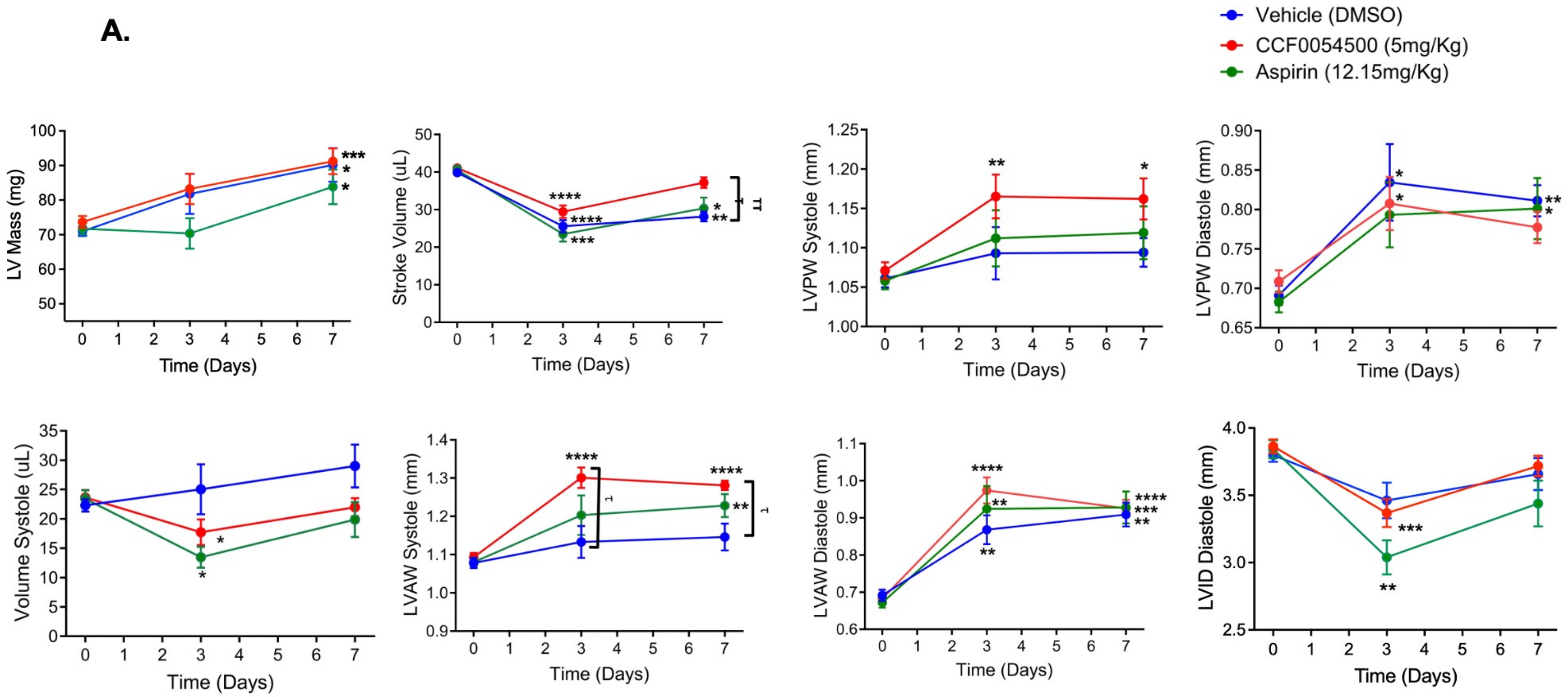
A.
Vehicle (DMSO)
CCF0054500 (5mg/Kg)
Aspirin (12.15mg/Kg)
LV Mass (mg)
Stroke Volume (uL)
LVPW Systole (mm)
LVPW Diastole (mm)
Volume Systole (uL)
LVAW Systole (mm)
LVAW Diastole (mm)
LVID Diastole (mm)
Time (Days)

B.

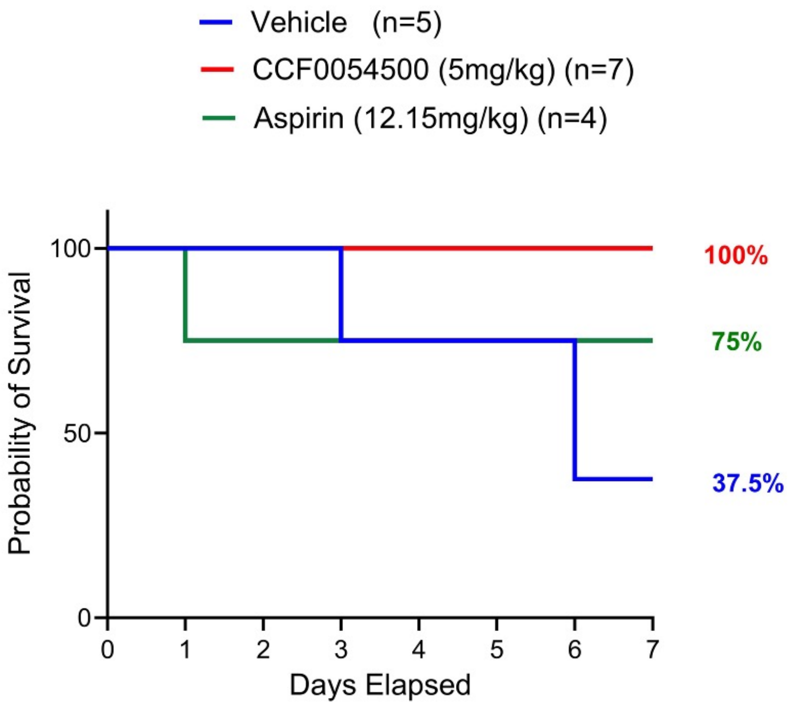
Vehicle (n=5)
CCF0054500 (5mg/kg) (n=7)
Aspirin (12.15mg/kg) (n=4)
100
50
0
Probability of Survival
0
1
2
3
4
5
6
7
Days Elapsed
100%
75%
37.5%

c.

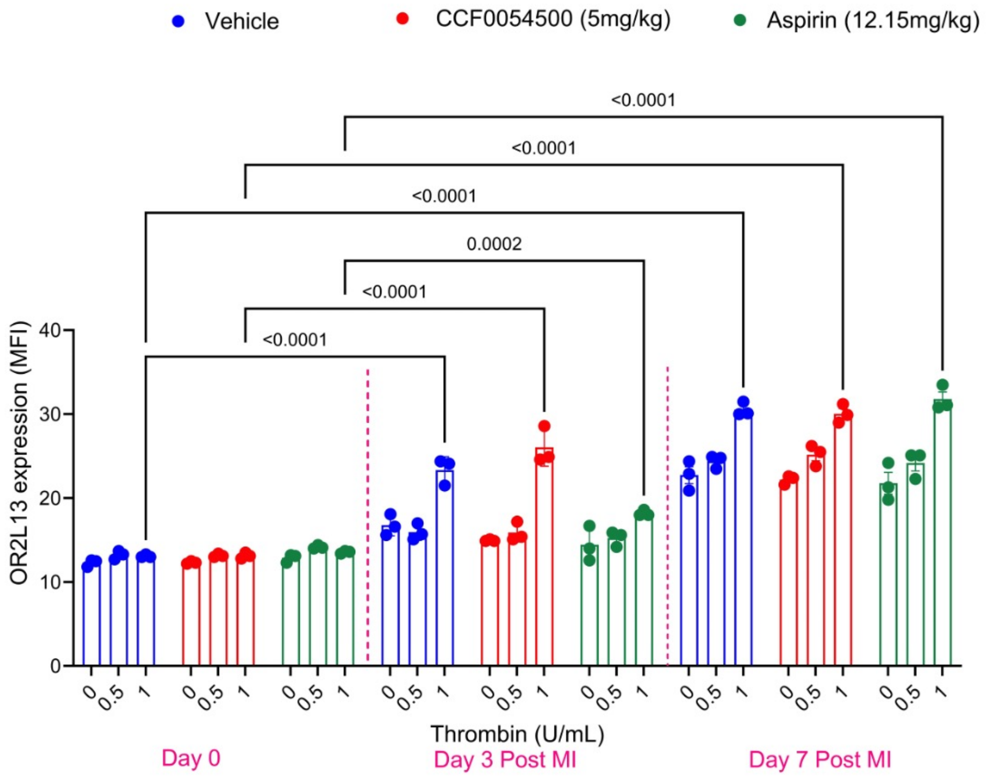
Vehicle
CCF0054500 (5mg/kg)
Aspirin (12.15mg/kg)
<0.0001
<0.0001
<0.0001
0.0002
<0.0001
<0.0001
OR2L13 expression (MFI)
40
30
20
10
0
0
0.5
1
Thrombin (U/mL)
Day 0
Day 3 Post MI
Day 7 Post MI

D.

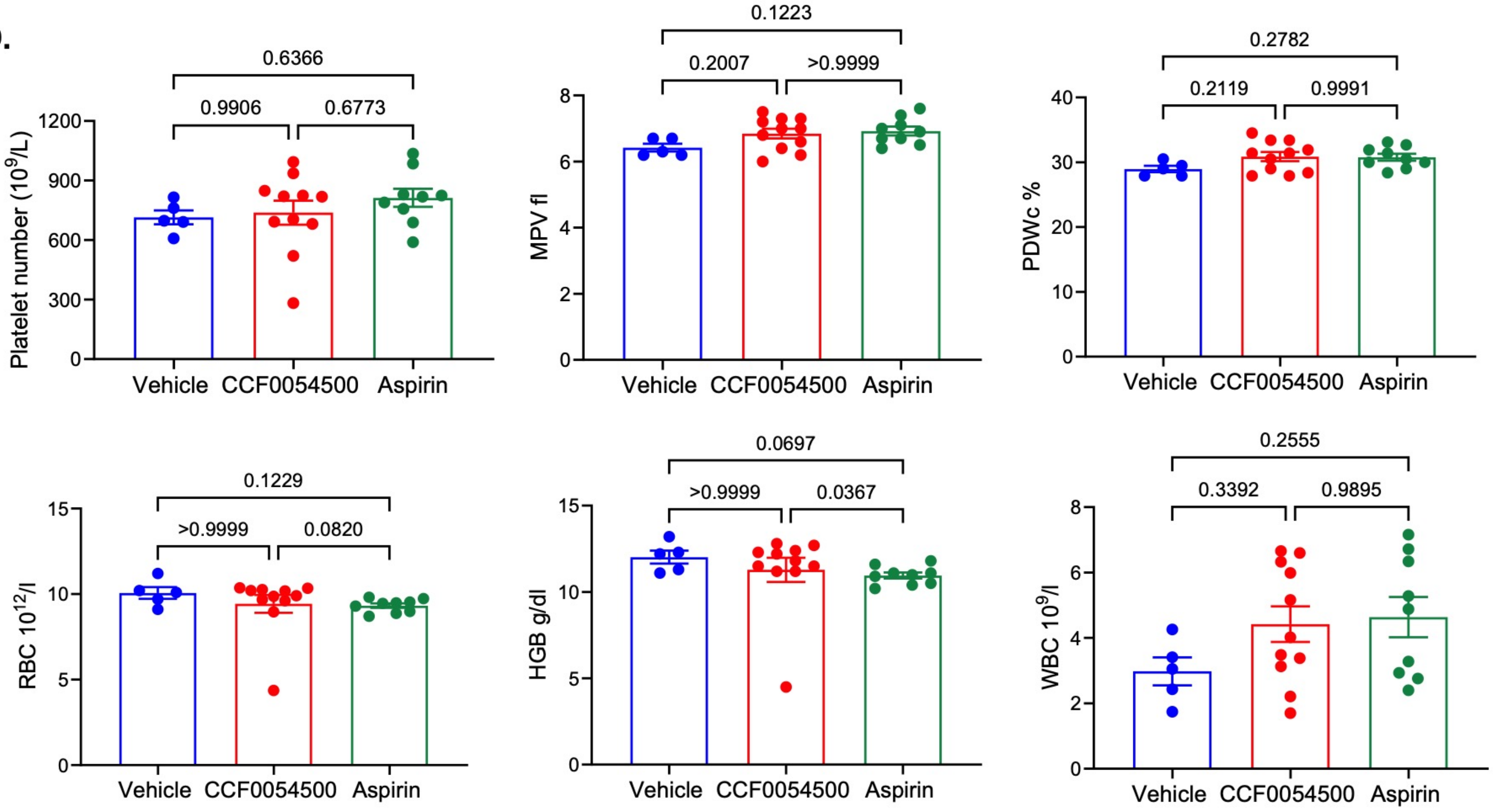

Figure S8: Effect of CCF0054500 on Platelet reactivity and cardiac function after Myocardial Infarction in mice. Left anterior descending (LAD) coronary aorta of FVB mice was ligated to give these mice myocardial infarction (n=6 in each group). Then these mice were injected with Vehicle (75% DMSO), CCF0054500 (5mg/kg per day) or Aspirin (12.15mg/kg per day) for seven days.

(A). LV Function measured as different parameters. P-Value is determined by Two-way ANOVA with repeated measures and Tukey post-hoc test relative to Day 0 for that treatment group. *, $P < 0.05$; **, $P < 0.01$; ***, $P < 0.01$; ****, $P < 0.0001$. ꚍ, $P < 0.05$; ꚍꚍ, $P < 0.01$; ꚍꚍꚍ, $P < 0.001$ by Two-way ANOVA with repeated measures and Tukey post-hoc test relative to vehicle control at a given time point. LV Mass-Left Ventricular Mass, LVAW- LV Anterior wall thickness, LVPW- LV Posterior wall thickness, LVID- LV Internal Diameter.

(B). Survival analysis: Kaplan-Meier (KM) curve shows the survival of the vehicle-treated (n=5), CCF0054500-treated (n=7) and aspirin-treated (n=4) mice. CCF0054500 improved the life expectancy post MI by 100%, and Aspirin improved life expectancy by 75%. A downward deflection signifies death. **(C).** OR2L13 expression after MI. Blood was collected at Day 0 (before the LAD ligation), Day 3 and Day 7 (Post-MI) from retro-orbital sinus. Platelets were isolated and stained with OR2L13 antibody for 30 minutes at room temperature to measure the expression of surface OR2L13 with the progression of MI. The results are expressed as Mean Fluorescence of OR2L13 (n=6 in each group). Data was analyzed using two-way ANOVA with Sidak's multiple comparisons test to compare treatment groups at each thrombin concentration. **(D).** No effect of CCF0054500 (5mg/kg per day) and Aspirin (12.15mg/kg per day) on thrombopoiesis, leukopoiesis, or hematopoiesis. Statistical analysis between groups was performed according to One way ANOVA or Kruskal Wallis test as appropriate. MPV=Mean Platelet Volume, RBC=red blood cells, HGB= Hemoglobin, PDW=Platelet Distribution Width, WBC=white blood cells.

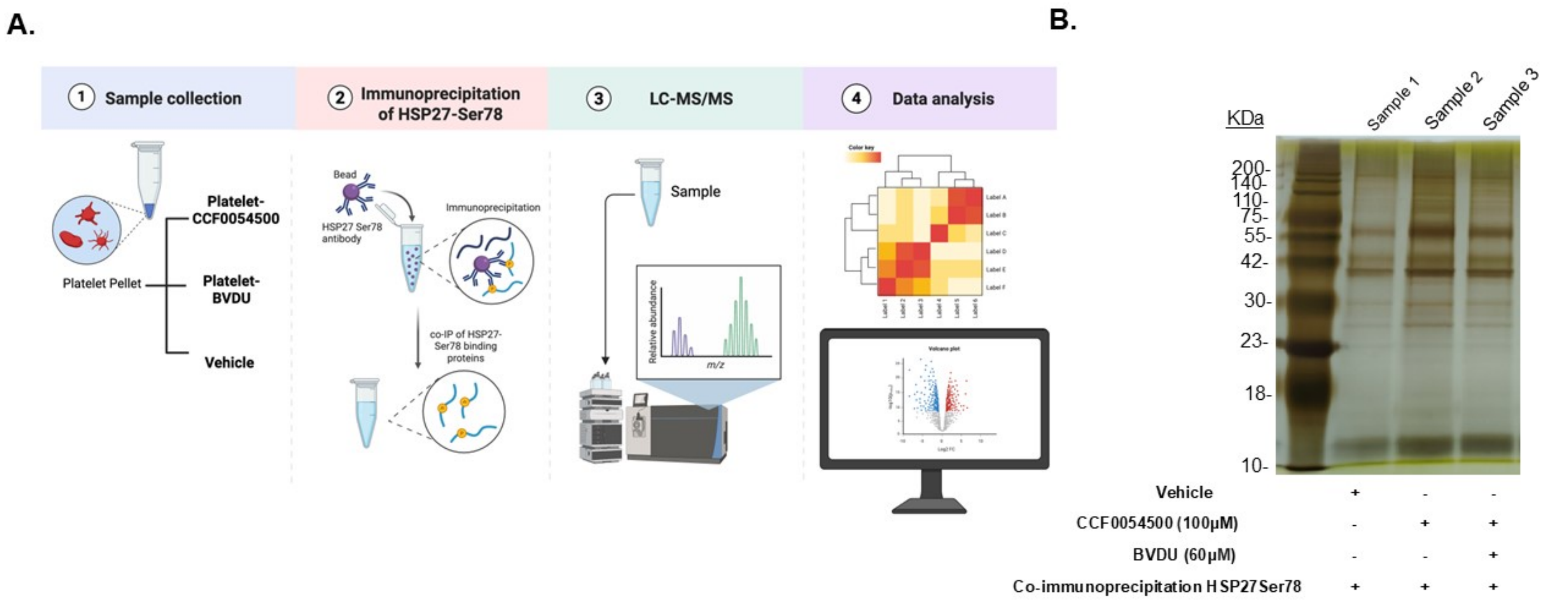

Figure S9: CCF0054500 rearranges the platelet actin cytoskeleton through HSP27. (A). Experimental workflow for determining the signaling pathways in the platelets that lead to decreased platelet reactivity through OR2L13 activation by CCF0054500. **(B).** Silver nitrate staining of isolated platelet proteins following SDS-PAGE with HSP27-Ser78 pulldown, with and without the BVDU (HSP27 inhibitor)

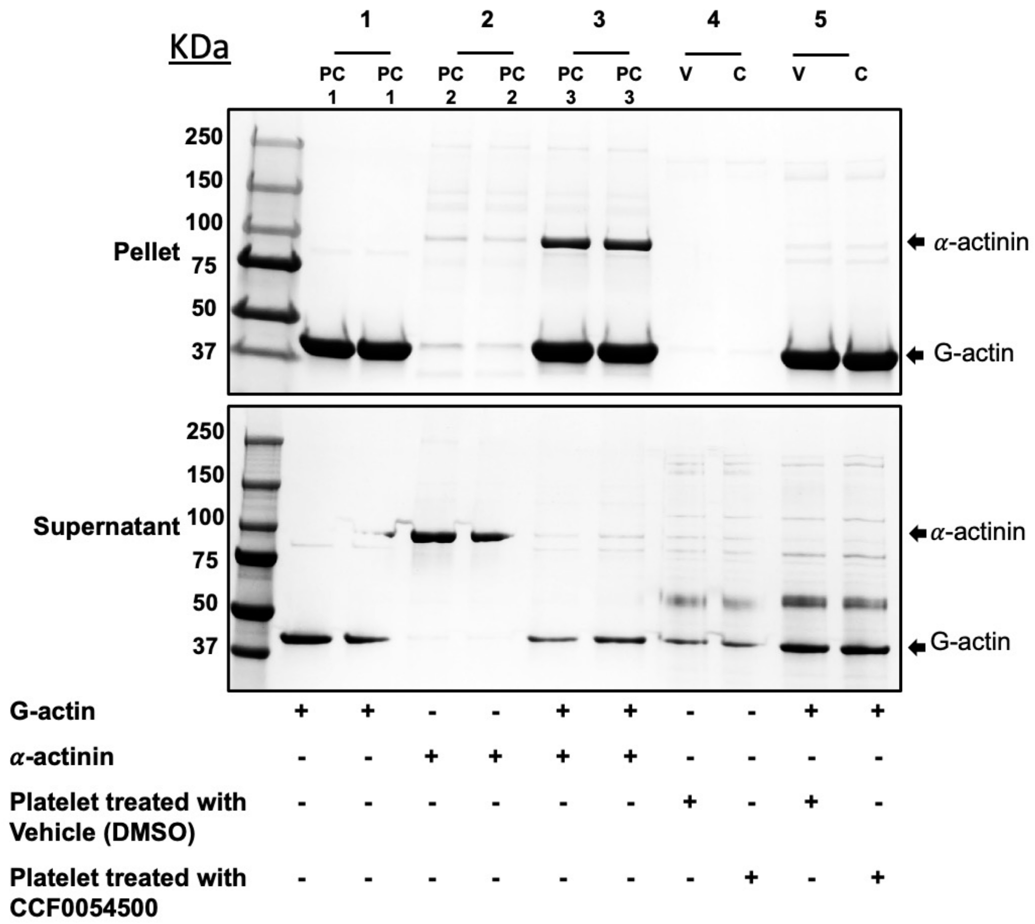

Figure S10: G-actin binding assay. G-actin binding assay was performed on washed platelets treated with vehicle or 100µM CCF0054500 according to method 2 of Actin Binding Protein Biochem kit from Cytoskeleton, Inc. Samples of the Pellet and supernatant fractions were collected for each reaction and were separated on 4-20% SDS-gel and stained with 0.1% Coomassie blue. Lane 1, G-actin (positive control 1). Lane 2, α-actinin (positive control 2). Lane 3, α-actinin and G-actin (positive control 3). Lane 4, Test sample alone. Lane 5, Test sample and G-actin. In Lane 5, in the presence of G-actin, there is no change in test protein of the pellet from CCF0054500 treated washed platelets compared to vehicle, suggesting no depletion of G-actin pool with CCF0054500. PC1-3 =Positive control 1-3, V= Vehicle and C=CCF0054500.

A.

Targets:

ADORA2A-Calcium Flux-Agonist
ADORA2A-Calcium Flux-Antagonist
ADRA1A-Calcium Flux-Agonist
ADRA1A-Calcium Flux-Antagonist
ADRA2A-cAMP-Agonist
ADRA2A-cAMP-Antagonist
ADRB1-cAMP-Agonist
ADRB1-cAMP-Antagonist
ADRB2-cAMP-Agonist
ADRB2-cAMP-Antagonist
AVPR1A-Calcium Flux-Agonist
AVPR1A-Calcium Flux-Antagonist
CCKAR-Calcium Flux-Agonist
CCKAR-Calcium Flux-Antagonist
CHRM1-Calcium Flux-Agonist
CHRM1-Calcium Flux-Antagonist
CHRM2-cAMP-Agonist
CHRM2-cAMP-Antagonist
CHRM3-Calcium Flux-Agonist
CHRM3-Calcium Flux-Antagonist
CNR1-cAMP-Agonist
CNR1-cAMP-Antagonist
CNR2-cAMP-Agonist
CNR2-cAMP-Antagonist
DRD1-cAMP-Agonist
DRD1-cAMP-Antagonist
DRD2S-cAMP-Agonist
DRD2S-cAMP-Antagonist
EDNRA-Calcium Flux-Agonist
EDNRA-Calcium Flux-Antagonist
HRH1-Calcium Flux-Agonist
HRH1-Calcium Flux-Antagonist
HRH2-cAMP-Agonist
HRH2-cAMP-Antagonist
HTR1A-cAMP-Agonist
HTR1A-cAMP-Antagonist
HTR1B-cAMP-Agonist
HTR1B-cAMP-Antagonist
HTR2A-Calcium Flux-Agonist
HTR2A-Calcium Flux-Antagonist
HTR2B-Calcium Flux-Agonist
HTR2B-Calcium Flux-Antagonist
OPRD1-cAMP-Agonist
OPRD1-cAMP-Antagonist
OPRK1-cAMP-Agonist
OPRK1-cAMP-Antagonist
OPRM1-cAMP-Agonist
OPRM1-cAMP-Antagonist
CAV1.2-Ion Channel-Blocker
GABAA-Ion Channel-Blocker
GABAA-Ion Channel-Opener
hERG-Ion Channel-Blocker
HTR3A-Ion Channel-Blocker
HTR3A-Ion Channel-Opener
KvLQT1/minK-Ion Channel-Blocker
KvLQT1/minK-Ion Channel-Opener
nAChR(a4/b2)-Ion Channel-Blocker
nAChR(a4/b2)-Ion Channel-Opener
NAV1.5-Ion Channel-Blocker
NMDAR (1A/2B)-Ion Channel-Blocker
NMDAR (1A/2B)-Ion Channel-Opener
INSR-Binding-Inhibitor
LCK-Binding-Inhibitor
ROCK1-Binding-Inhibitor
VEGFR2-Binding-Inhibitor
AR-NHR Nuclear Translocation-Agonist
AR-NHR Nuclear Translocation-Antagonist
GR-NHR Protein Interaction-Agonist
GR-NHR Protein Interaction-Antagonist
AChE-Enzymatic-Inhibitor
COX1-Enzymatic-Inhibitor
COX2-Enzymatic-Inhibitor
MAOA-Enzymatic-Inhibitor
PDE3A-Enzymatic-Inhibitor
PDE4D2-Enzymatic-Inhibitor
DAT-Transporter-Blocker
NET-Transporter-Blocker
SERT-Transporter-Blocker

B.

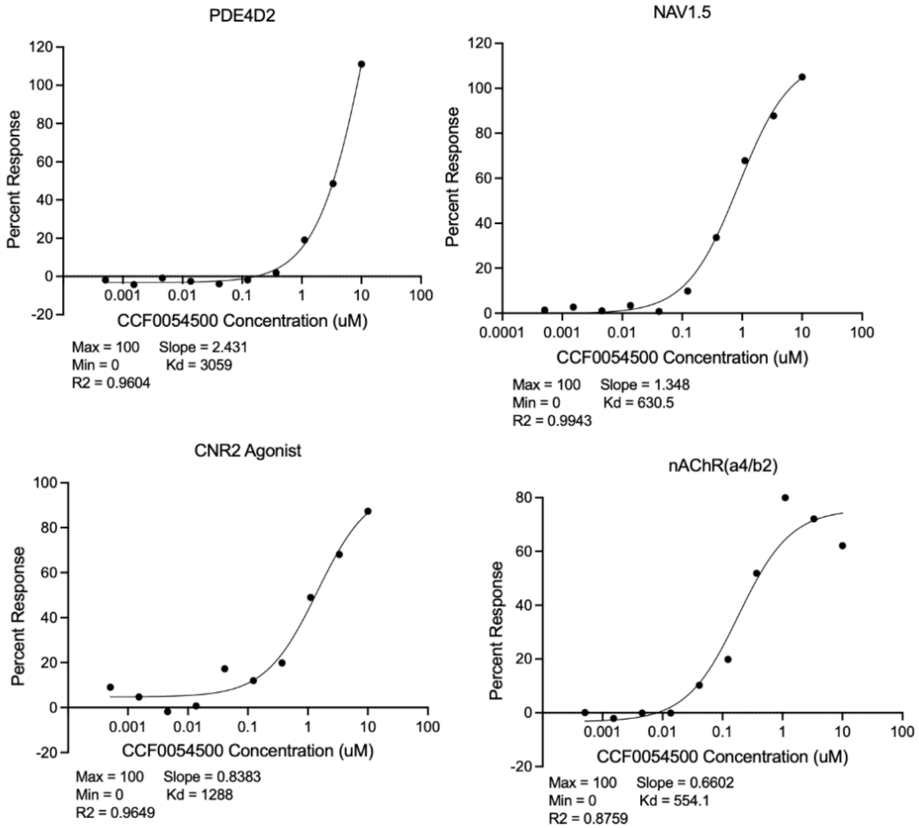

C.

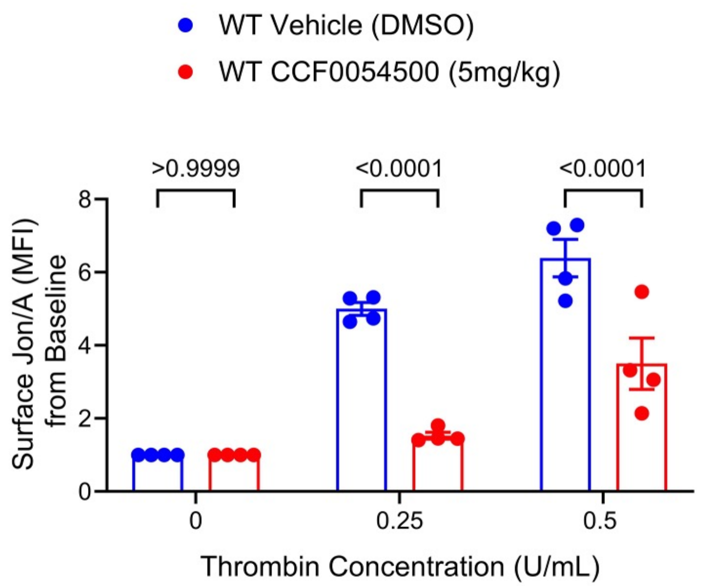

D.

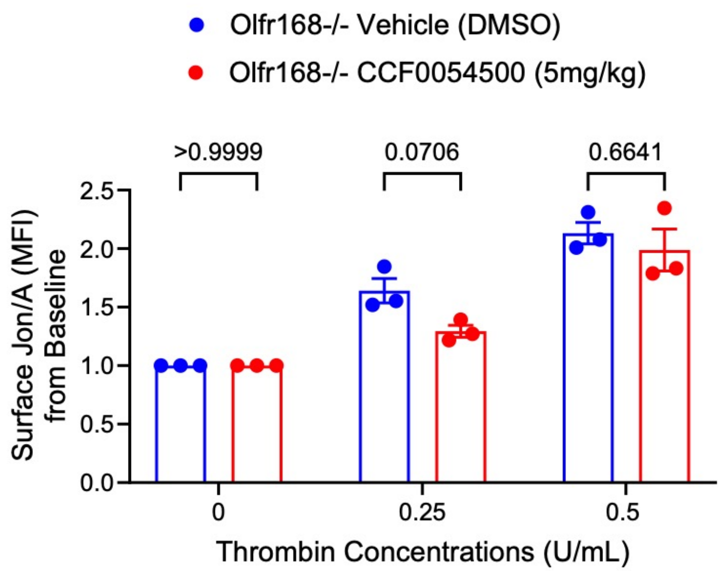

Figure S11: Off-target effects of CCF0054500. (A). Eurofins off-target analysis of CCF0054500 using cell lines expressing GPCRs, nuclear receptors, ion channels and enzymes. 78 Assays tested are indicated above. Off-target effects were noted for a sodium channel, a phosphodiesterase isoform, and the nicotinic acetyl choline receptor, and cannabinoid Receptor 2 (log dose-response curves indicated). All other targets were negative for cross-reactivity. **(B).** Accompanying Excel file is uploaded with raw pharmacological data. CCF0054500 in-vivo effects. **(C).** WT and **(D).** *Olfr168-/-* mice were injected with Vehicle (75% DMSO) or CCF0054500 (5mg/Kg per day) IP X 3 days. The blood was collected from retro-orbital sinus on Day 3, and platelets were isolated. The platelets were stimulated with Thrombin (0, 0.25, 0.5 U/mL) for 15 minutes and then stained for Jon/A. The results are expressed as Mean Fluorescence of Jon/A from the baseline (n=4, Two-way ANOVA). Changes in Surface Jon/A expression provides information about platelet aggregation.

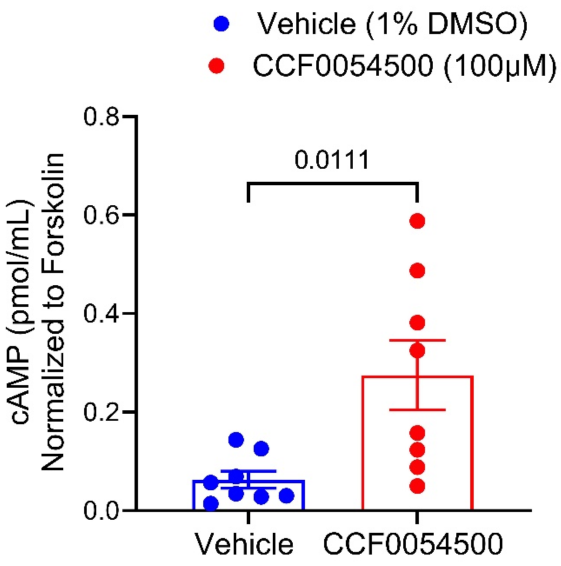

Figure S12: CCF0054500 generates cAMP in platelets. Platelets were treated with vehicle (DMSO), 100 µM CCF0054500, and 5µM forskolin (positive control) for 5 minutes. cAMP was measured during the Direct cAMP ELISA kit (Enzo). Data was normalized to forskolin for each subject. Data are represented as mean ± SEM, n=8 (unpaired t-test).

Videos S1: Intravital microscopy video of mouse cremaster arterioles showing platelet (green) and fibrin (red) accumulation following laser injury in vehicle treated mice

Video S2: Intravital microscopy video of mouse cremaster arterioles showing platelet (green) and fibrin (red) accumulation following laser injury in CCF0054500 treated mice

Excel File S1: Differentially modified phosphoproteins identified following platelets treated with vehicle or CCF0054500 (Dataset for Figure 7A).

Excel File S2: Differentially expressed phosphoproteins identified following co-immunoprecipitation of platelets treated with vehicle, CCF0054500, or CCF0054500 + BVDU (HSP27 inhibitor), using an HSP27-Ser78 antibody (Dataset for Figure 7H).
