# Supplementary material for: Olfactory Receptor Activation Reduces Platelet Reactivity and Arterial Thrombosis Through Actin Cytoskeleton Remodeling
Source: Circulation. 2026 Apr 7;153(23):1827–44. doi: 10.1161/CIRCULATIONAHA.125.078927 (PMC13236044; doi:10.1161/CIRCULATIONAHA.125.078927)
Supplement: Supplementary file 6 [file cir-153-1827-s006.pdf]

Figure 7D in the manuscript

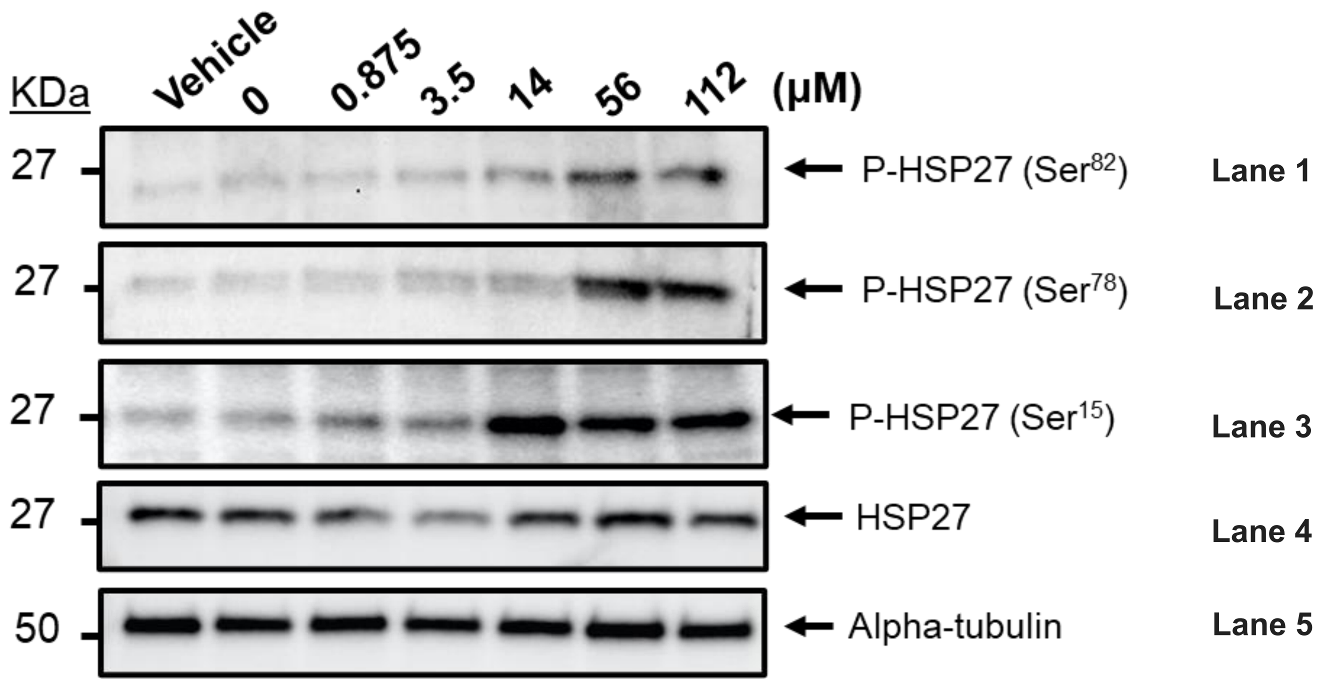

Full unedited gel for Figure 7D

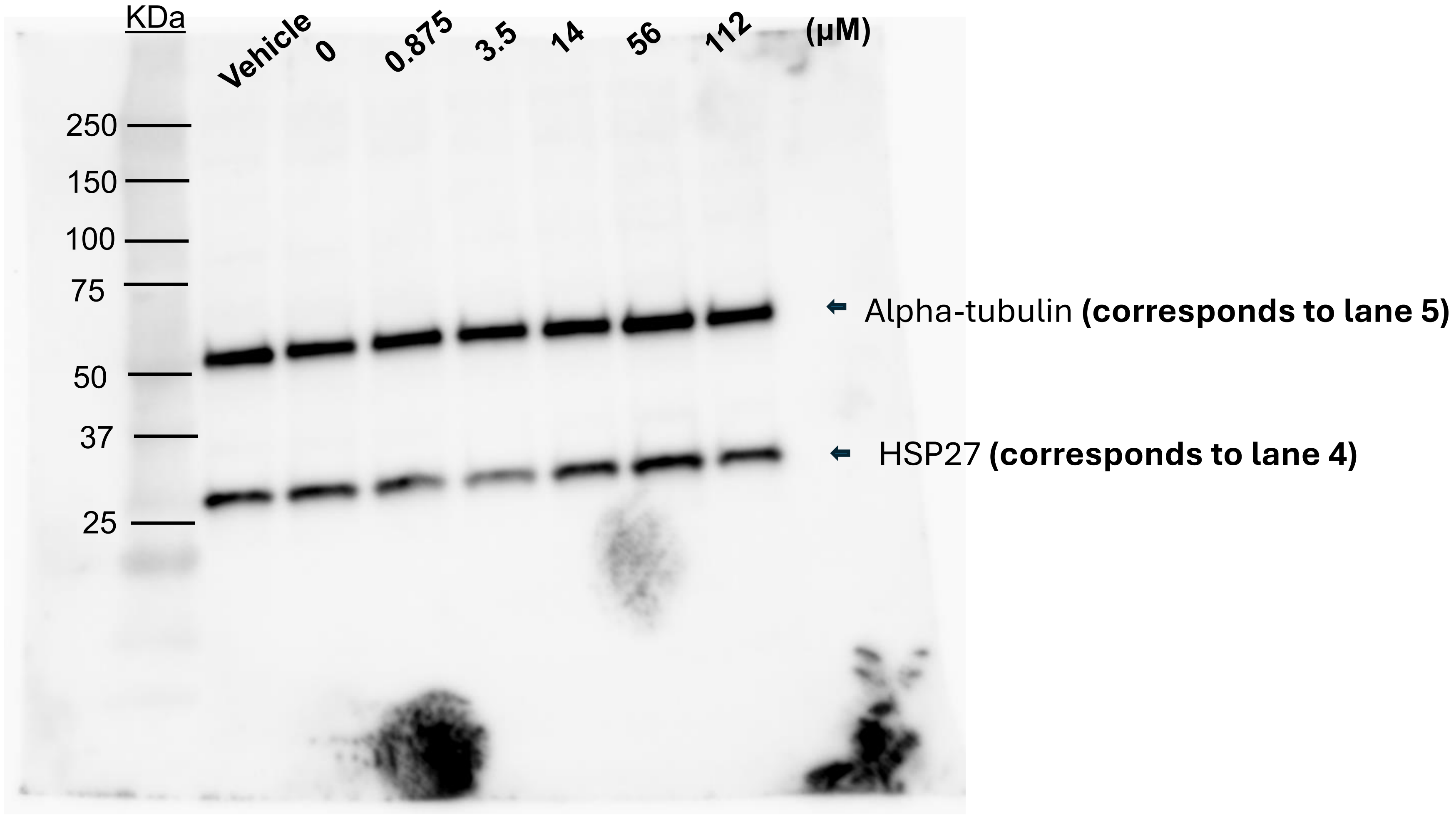

Full unedited gel for Figure 7D

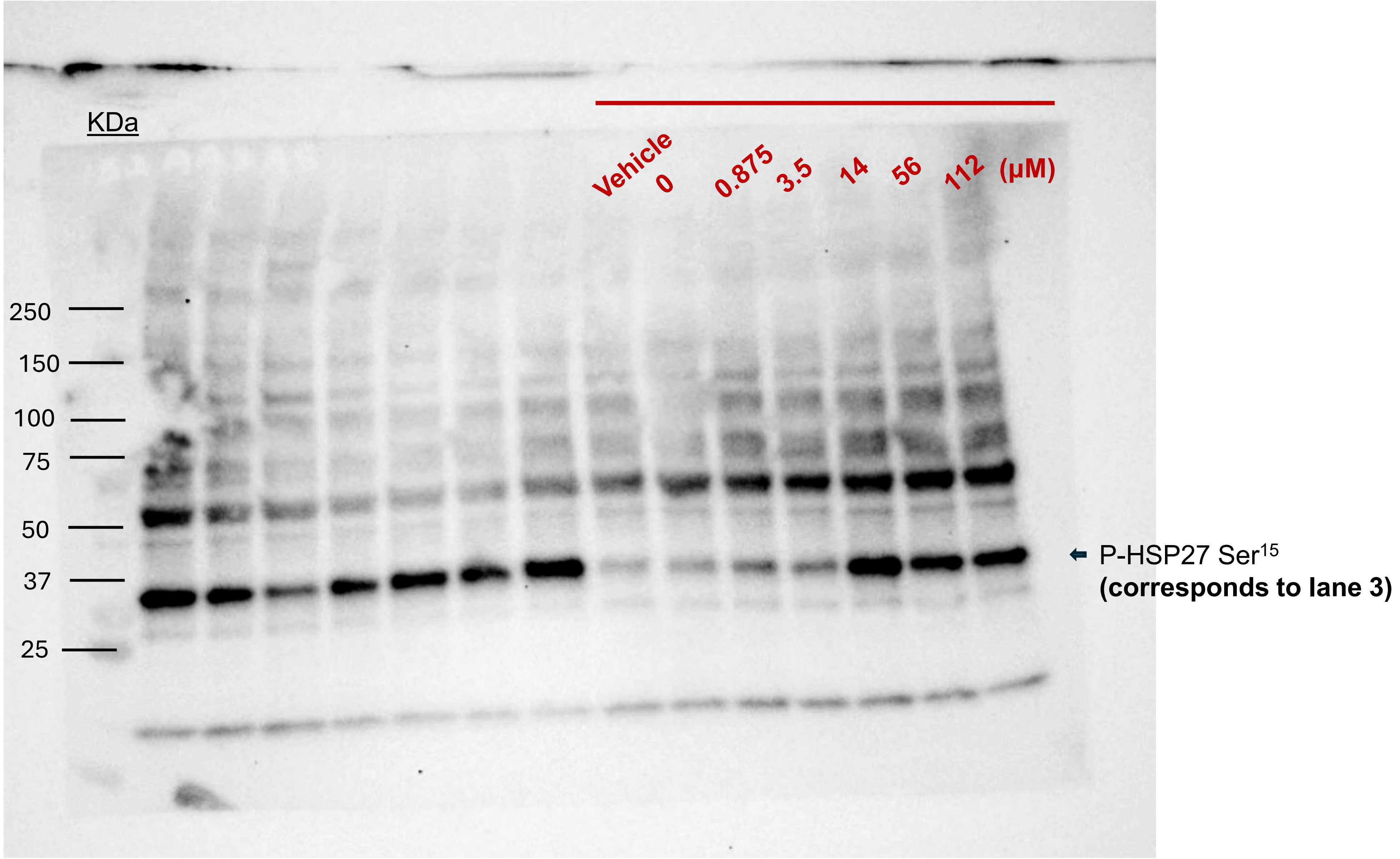

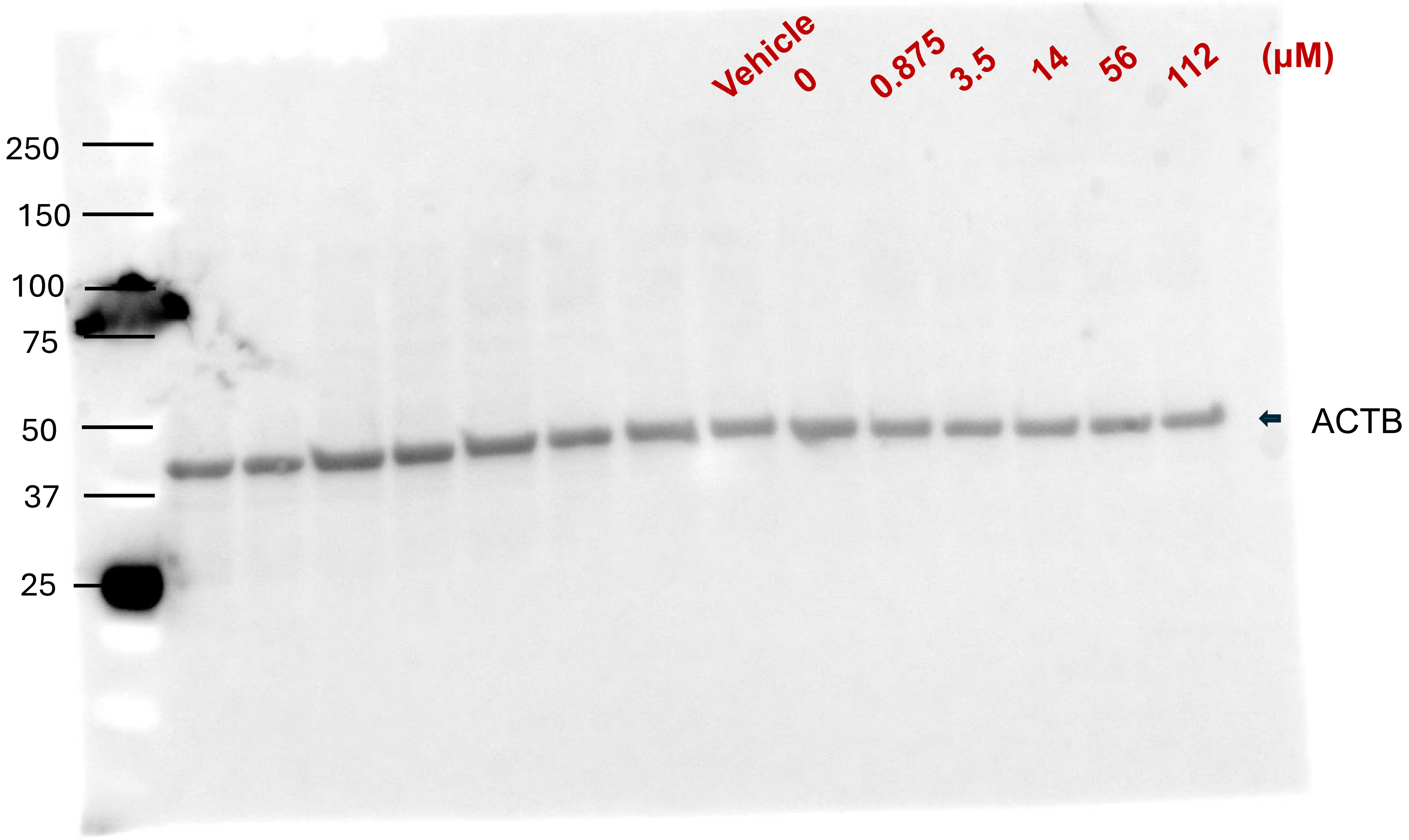

(loading control for P-HSP27 Ser[15])

Full unedited gel for Figure 7D

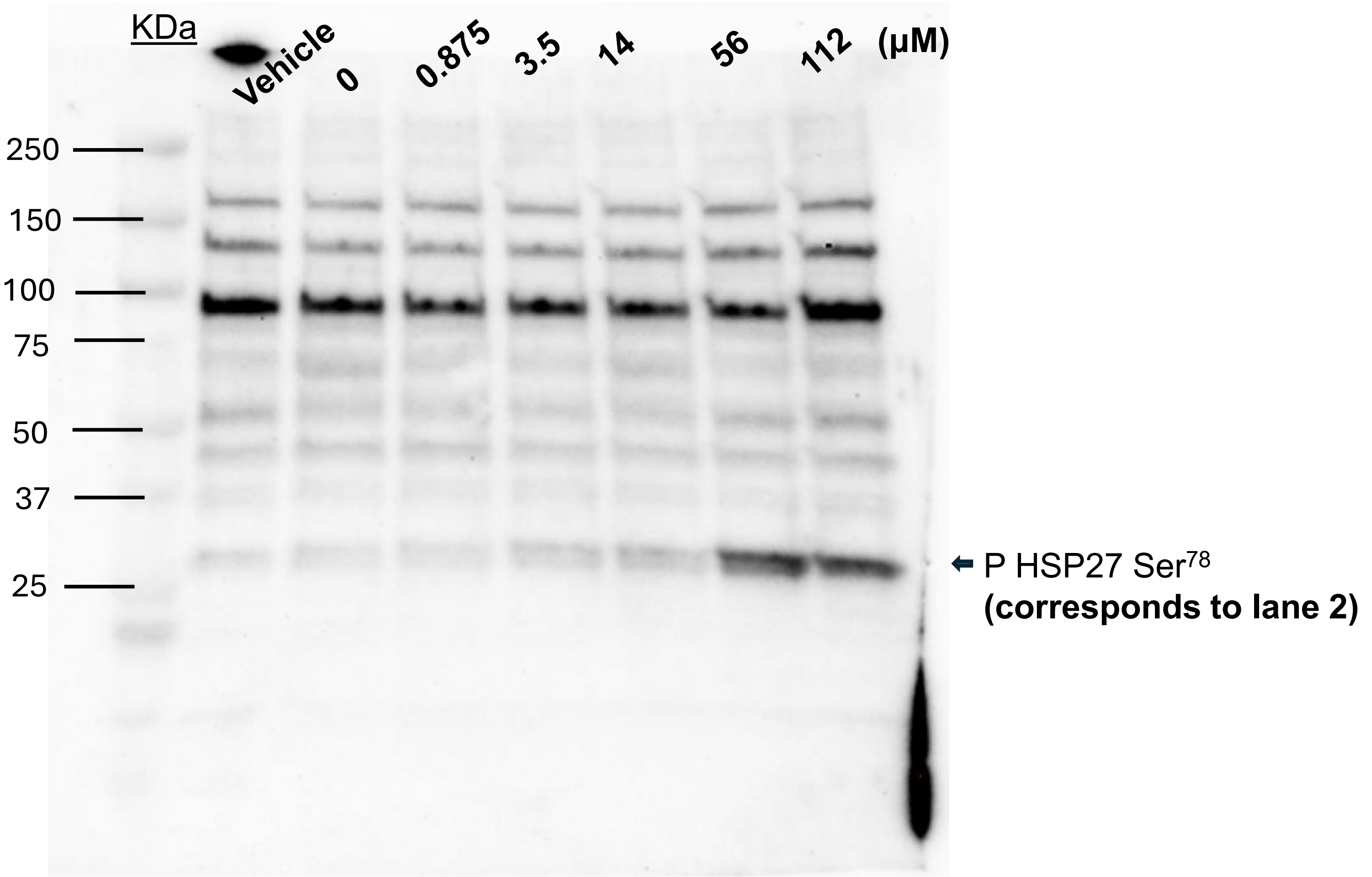

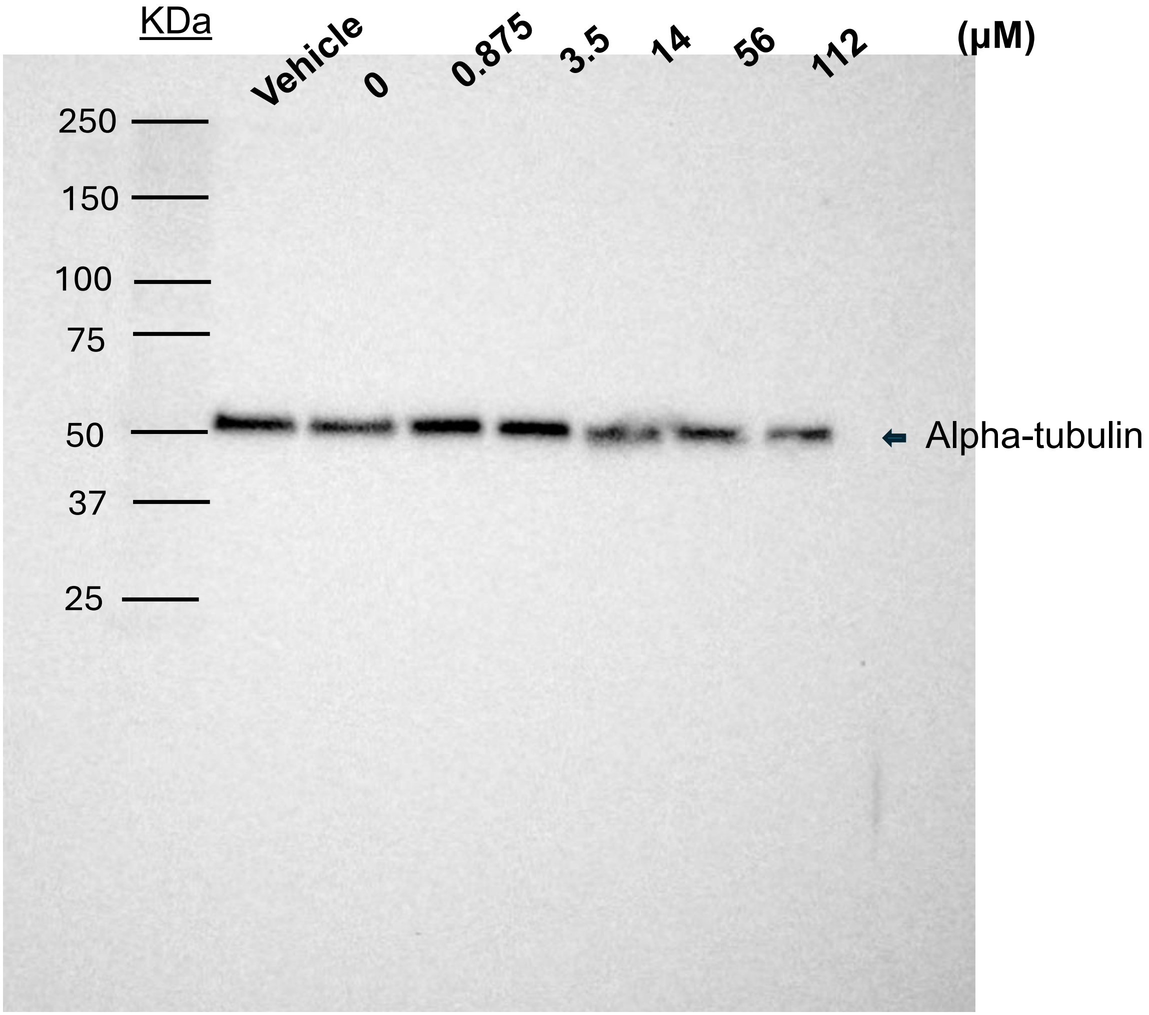

(loading control for P-HSP27 Ser^{78})

Full unedited gel for Figure 7D

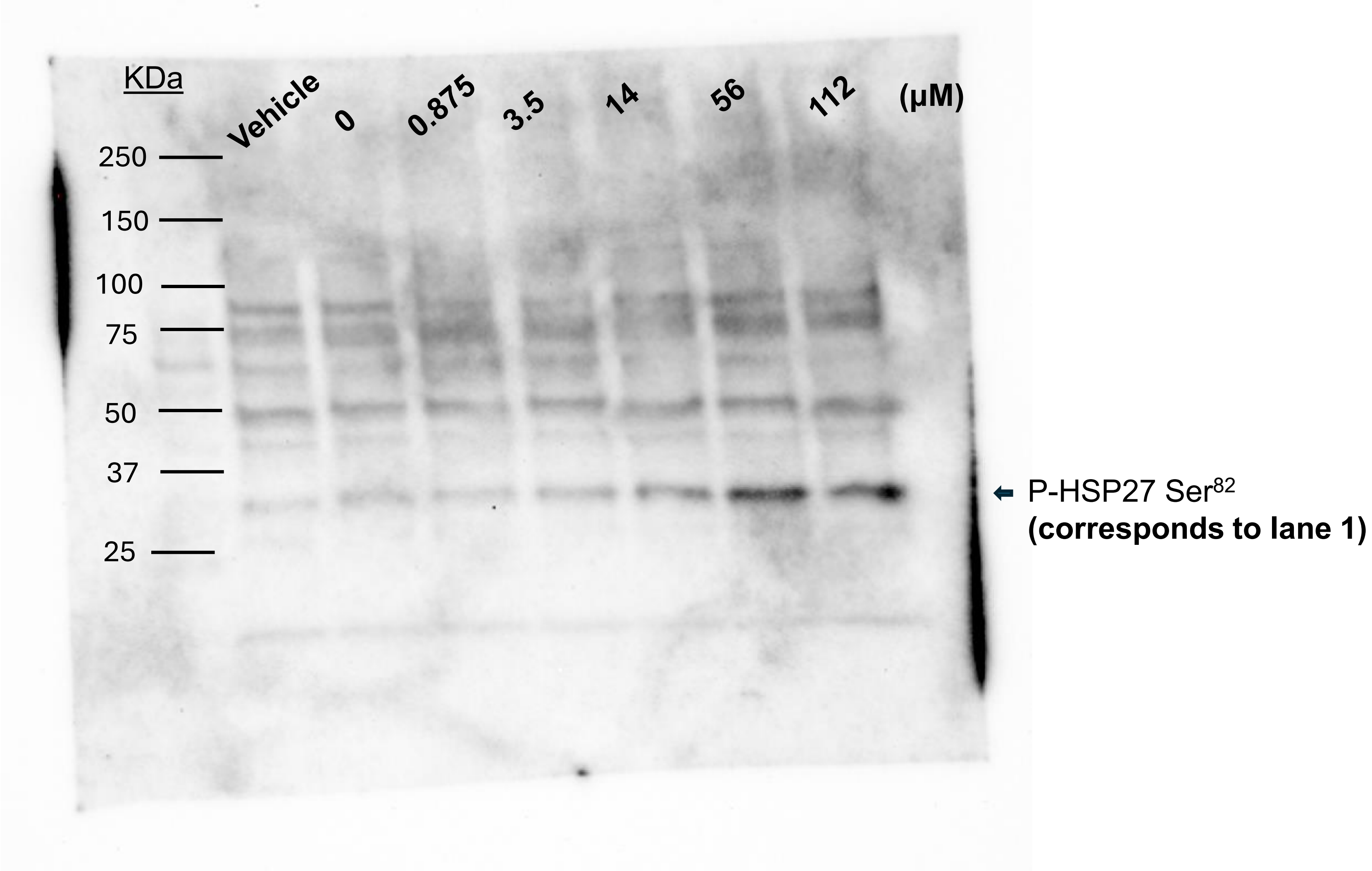

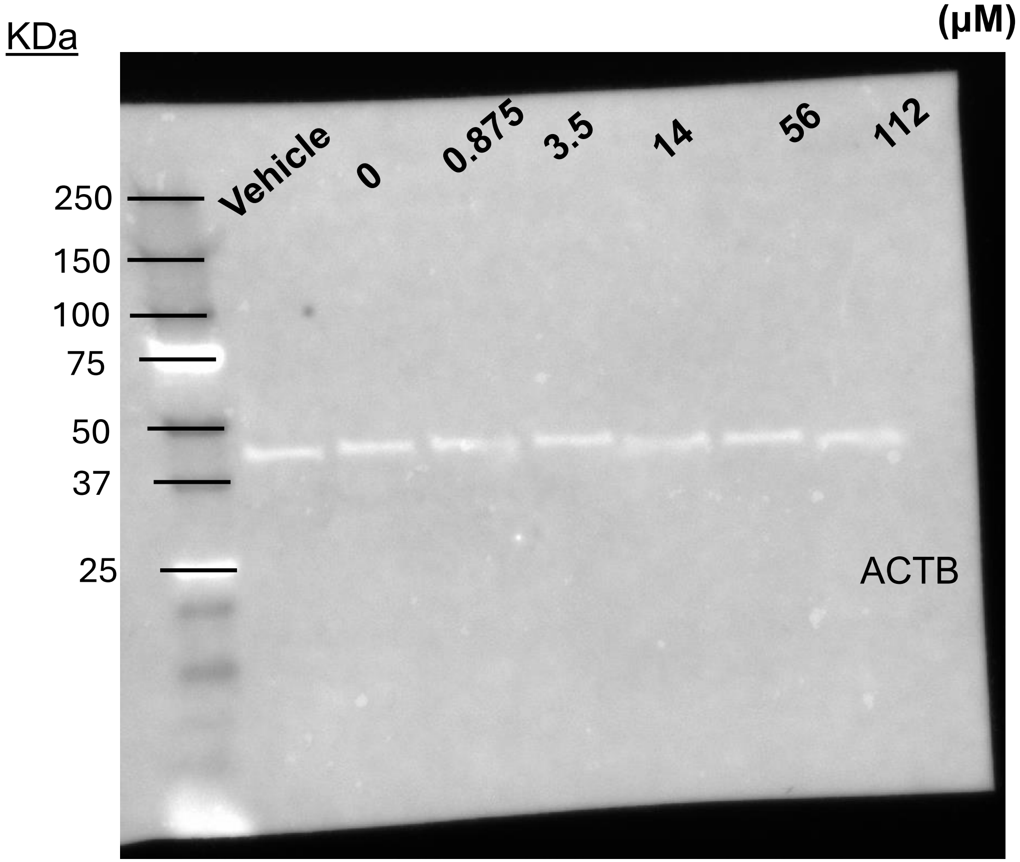

(loading control for P-HSP27 Ser[82])
